# From genomic discovery to application in age-related hearing loss: a global bibliometric and cross-ethnic analysis

**DOI:** 10.3389/fnagi.2025.1678115

**Published:** 2025-11-03

**Authors:** Yang Lu, Jiawei Shen, Ka Ho Kairos Sou, Hsi Lu, Shuoyuan Huang, Kai Uus

**Affiliations:** ^1^Division of Psychology, Communication and Human Neuroscience, The University of Manchester, Manchester, United Kingdom; ^2^Department of Speech and Hearing Rehabilitation Science, East China Normal University, Shanghai, China; ^3^Manchester Centre for Audiology and Deafness, School of Health Sciences, The University of Manchester, Manchester, United Kingdom

**Keywords:** age-related hearing loss (ARHL), genetics, bibliometric analysis, cross-ethnic, candidate genes

## Abstract

**Introduction:**

Age-related hearing loss (ARHL) is a common chronic condition that significantly affects the quality of life in older adults. Studies have shown that genetic factors play a substantial role in ARHL, with heritability estimates ranging from 46 to 74%. Although advances in genomics and epigenetics have led to the identification of numerous candidate genes in recent years, most related studies have focused on European and North American populations. There remains a lack of systematic mapping of research trends and cross-ethnic gene consistency, limiting the broad applicability of these findings.

**Method:**

This study screened English-language publications on ARHL genetics from 1995 to June 2025 across PubMed, Embase, Web of Science, and Scopus, ultimately including 465 studies. Bibliometric analyses were conducted using R Bibliometrix, VOSviewer, and CiteSpace to extract research trends, research hotspots, and candidate genes. Ethnic information from human studies were compiled to facilitate cross-ethnic comparative analysis.

**Result:**

Over the past 30 years, publications in this field have shown continuous growth, with an average annual growth rate of 6.83%. Hearing Research emerged as the core journal. China and the United States were the top two publishing countries, though international collaboration remained limited. Research priorities have gradually shifted from inner ear anatomy to molecular mechanisms such as gene variants, oxidative stress, mitochondrial function, and inflammation. A total of 365 candidate factors from animal studies and 221 candidate genes from human studies were extracted and grouped into seven categories. Cross-ethnic analysis identified 56 genes that were repeatedly reported across at least two populations. Among these, *CDH23*, *ILDR1*, and *SLC26A5* showed high cross-ethnic consistency, while genes such as *GRHL2* exhibited notable ethnic specificity.

**Conclusion:**

This study systematically maps the developmental trajectory and research hotspots of ARHL genetics, revealing key patterns in geographic distribution, thematic evolution, and cross-ethnic applicability. The findings highlight the urgent need to strengthen research in non-European populations and promote international collaboration, thereby providing a theoretical foundation and data support for building a universally applicable genetic risk framework and advancing individualised interventions.

## 1 Introduction

With global ageing, age-related diseases have emerged as a major public health challenge, profoundly affecting the daily lives of older adults. As of 2021, the global population aged 65 years and older reached 761.2 million, accounting for 9.62% of the total world population. A UN report predicted that by 2050, this number will double to approximately 1.5 billion people (United Nations et al., 2019). Age-related hearing loss (ARHL) is the third most common chronic health condition among the elderly, following arthritis and cardiovascular disease (Palmer et al., 2017), affecting nearly two-thirds of adults over 70 years old (Lin F. R. et al., 2011). Also referred to as presbycusis, ARHL is defined as a progressive, bilateral, and symmetrical sensorineural hearing loss predominantly affecting higher frequencies (Bowl and Dawson, 2019). It typically begins around the age of 40, with a marked increase in prevalence among those aged 80 and above. The progression of ARHL is gradual and irreversible. ARHL has been closely associated with a spectrum of adverse health conditions, including accelerated cognitive decline, depression, increased risk of dementia, impaired balance, and falls. In addition, ARHL imposes significant social consequences such as impaired communication, social withdrawal, reduced autonomy, and diminished economic productivity, which compromise the quality of life in older adults (Davis et al., 2016; Loughrey et al., 2018). The ongoing rise in ARHL prevalence, driven by population ageing, has significantly heightened the medical and socioeconomic demands placed on public health infrastructures. ARHL has become a pressing public health concern requiring urgent attention and intervention.

The aetiology of ARHL is multifactorial, encompassing internal factors such as genetic susceptibility, epigenetic changes, and aging, as well as external factors such as noise exposure, use of ototoxic drugs, head trauma, and smoking history. These factors interactions drive the onset and progression of ARHL (Keithley, 2020). Genetic factors is particularly influential in the pathophysiological mechanisms of ARHL. Current heritability estimates indicate that genetic variation may explain between 46 and 74% of the variance in hearing thresholds among individuals with ARHL (Duan et al., 2019; Momi et al., 2015; Wolber et al., 2012). Genetic contributions may operate through diverse mechanisms, including mutations in chromosomal or mitochondrial genes, epigenetic dysregulation such as DNA methylation, and gene-environment interactions (Duan et al., 2019; Guo et al., 2023; Xu et al., 2021). For instance, mitochondrial DNA (mtDNA) mutations can impair inner ear energy metabolism, while alterations in genomic DNA methylation may affect the expression of genes involved in auditory function. Moreover, chronic noise exposure may provoke outer hair cell damage in individuals with a susceptible genetic background (Cornejo-Sanchez et al., 2025; Guo et al., 2023; Kujawa and Liberman, 2006; Wolber et al., 2014). These overlapping mechanisms jointly shape the heterogeneous characteristics of ARHL.

In recent years, significant advances have been made in uncovering the genetic landscape of ARHL, driven by the rapid development of genome-wide association studies (GWAS), DNA methylome analysis and multi-omics integration technologies. Numerous susceptibility loci have been identified, particularly in large-scale GWAS of European and North American populations. Genes such as *GRM7, LOXHD1, TRIOBP, FBF1*, and *PRKAG2* have been repeatedly associated with high frequency hearing loss, gradually constructing a core ARHL candidate gene repository (Friedman et al., 2009; Hoffmann et al., 2016; Polesskaya et al., 2025). For example, a large-scale meta-analysis on GWAS by Ivarsdottir et al. (2021) identified 21 novel genetic loci, including rare mutations in *LOXHD1* and structural variants in *FBF1*, both of which were significantly associated with increased risk of hearing deterioration. In animal models, Polesskaya et al. (2025) performed a GWAS across nearly 1,000 ENU-mutagenised mice and demonstrated that deletion of *Prkag2* significantly accelerated high-frequency hearing loss, providing direct molecular evidence for the involvement of energy metabolism pathways in ARHL pathogenesis. In addition, Wells et al. (2020) conducted a synthesis of multiple GWAS datasets and identified a set of single nucleotide polymorphisms (SNPs) recurrently associated with ARHL. These variants span mitochondrial genes, cell adhesion molecules, and members of the glutamate receptor family, further indicating the multifactorial and polygenic nature of the ARHL’s pathology. Currently, ARHL research at the epigenetic level is advancing. DNA methylation analysis has revealed that methylation levels at promoter regions of genes such as *TCF25* and *POLE* are significantly correlated with auditory function, suggesting that epigenetic modification may play a key regulatory role in the progression of ARHL (Patil et al., 2024; Wolber et al., 2014). Furthermore, multi-omics integration strategies have enabled the identification of novel molecular biomarkers, offering new theoretical and methodological frameworks for early detection, precise stratification, and personalised intervention in ARHL.

However, ARHL genetic research continues to face multiple challenges. Despite a steady increase in the volume of related research and the identification of various candidate susceptibility genes for ARHL in recent years, a systematic synthesis of prevailing research hotspots, developmental trends, and key scientific questions remains lacking. Specifically, there remains a limited understanding of the thematic evolution of research topics, the prioritisation of high-frequency genes, the impact of landmark publications, and the translational potential of studies targeting these candidate genes. Therefore, a comprehensive bibliometric analysis is necessary to reveal the field’s research structure, evolutionary trends and future development directions. This would provide theoretical support for in-depth exploration of the genetic mechanism and precise intervention of ARHL. In addition, current research is heavily skewed toward European-ancestry cohorts, with significantly fewer studies addressing non-European populations such as those from Asia, Africa, and Latin America. This geographic and ethnic bias limits the generalisability of current findings and constrains their clinical translatability across diverse populations. Many susceptibility genes and genetic variants identified in European cohorts have yet to be validated in other ethnic groups, making it difficult to construct a globally representative panel of candidate genes. Given that differences in genetic background among different populations may influence the effects of pathogenic variants, a cross-ethnic comparative study is necessary to reveal the genetic mechanisms of ARHL.

This study adopts a global perspective and employs bibliometric and thematic evolution analysis to systematically map the developmental trajectory and shifting research foci of ARHL genetics over the past three decades. It also synthesises evidence from high-frequency candidate genes across different ethnic groups to identify genes that are cross-ethnically validated. This aims to uncover inter-population variability in genetic architecture and effect sizes, shedding light on how such heterogeneity may influence mechanistic interpretation and the generalisability of findings. Through this approach, the study aims to lay a robust empirical and theoretical foundation for future interdisciplinary and trans-regional collaborations in precision hearing health management.

## 2 Materials and methods

### 2.1 Literature sources and search strategy

To ensure the comprehensiveness of literature coverage, we thoroughly searched for publications between January 1995 to June 2025 spanning four databases: PubMed and Ovid Embase to cover biomedical and health sciences literature, and Web of Science Core Collection (WoS CC) and Scopus to capture broader scientific output. Search strategies were formulated with reference to previously published studies reporting findings closely related to the genetic factors of ARHL (Jung et al., 2024; Ninoyu and Friedman, 2024; Wells et al., 2020). The following retrieval strategy was employed: TS = [(“Presbycusis” OR “Age-related hearing loss” OR “ARHL”) AND “Genetics”] (see Supplementary Table 1 for database-specific, detailed search strategies).

### 2.2 Literature screening

**FIGURE 1:**
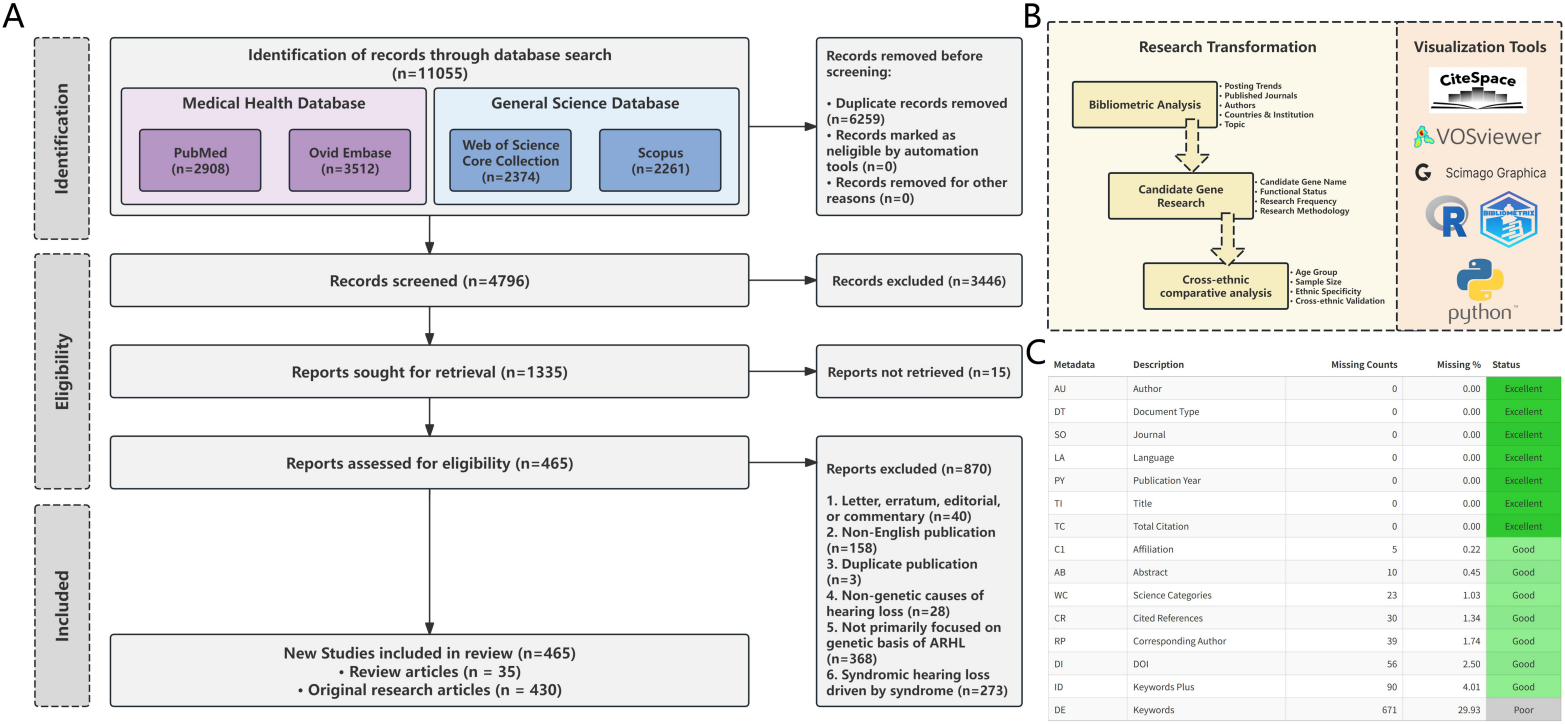
Overview of literature sources and research. **(A)** Literature screening flowchart. **(B)** Analytical structure of the study. **(C)** Literature Characterisation and Information Completeness.

All literature searches and data downloads were completed on October 1, 2024 from the four selected databases, gathering a total of 11,055 records. Duplicates were removed using EndNote 21 tool, followed by a manual review to remove any remaining duplicates. Two researchers with domain expertise (HL and KS) independently performed both the initial and secondary screening based on pre-defined inclusion and exclusion criteria (see Supplementary Table 2 for details). Discrepancies during the screening process were resolved through discussion with a third reviewer (YL) until consensus was reached. Ultimately, 465 articles demonstrating good inter-rater reliability (Kappa = 0.74) were included for analysis, comprising 430 original research articles and 35 reviews. Inter-rater reliability was assessed using Cohen’s kappa coefficient (κ). This metric was designed to account for the possibility of agreement occurring by chance, thereby providing a more robust measure of consensus. According to established benchmarks (El Emam, 1999), the resulting kappa value of κ = 0.74 indicated “substantial agreement”. This result confirmed a high degree of reliability in the judgments made by the reviewers in this study. The study selection process is illustrated in Figure 1A.

### 2.3 Data extraction and analysis

Bibliometric analysis and cross-ethnic comparative analysis were employed to examine the included literature (Figure 1B). In the bibliometric phase, comprehensive metadata were extracted, including title, abstract, keywords, authors, author affiliations/countries, year of publication, and journal name. Data processing and bibliometric analyses were conducted using the Bibliometrix package in R (Aria and Cuccurullo, 2017). The types of data analysed included key bibliometric variables such as author information, keywords, citation patterns, and the distribution of countries and institutions. The purpose of these analyses was to reveal research trends, identify core authors, and characterise the distribution of highly influential publications. Statistical visualisations were generated using ggplot2 in R and matplotlib in Python to illustrate publication trends, collaboration networks, and thematic evolution. In addition, VOSviewer, CiteSpace, and Scimago Graphica were employed for cluster analysis and network structure visualization (Chen, 2017; Hassan-Montero et al., 2022). The completeness of the bibliographic metadata used for bibliometric analysis is shown in Figure 1C.

### 2.4 Candidate gene analysis

A secondary extraction of candidate gene data was conducted from the included studies to enable further analysis. Studies were categorised by research subject into animal-based and human-based investigations. For each study, the candidate gene name, its function, and reporting frequency were extracted. In human studies, additional variables such as study methodology, participant age, and ethnic background were also recorded to capture heterogeneity in gene-level findings and study design. Based on these extracted variables, descriptive analyses were performed to summarise the distribution, frequency, and functional categorisation of candidate genes, providing a structured foundation for subsequent cross-ethnic comparative analysis.

### 2.5 Cross-ethnic comparison and visualisation of candidate genes

To explore both the convergence and divergence of candidate gene findings across populations, we conducted a cross-ethnic comparative analysis. Using ethnicity data extracted from human studies, we identified candidate genes reported in two or more distinct geographic populations, forming a subset of shared cross-population genes.

We employed the UpsetR package in R to visualise gene-level intersections among major population groups in Europe, North America, East Asia, South Asia, South America, and Africa. Using pheatmap in R, we further mapped the distribution of these shared genes, providing a structured overview of gene presence across regions. Together, these visualisations enabled the identification of candidate genes consistently reported across populations, while also offering a framework to disentangle region-specific patterns from globally recurrent signals, thereby advancing understanding of both genetic heterogeneity and cross-ethnic commonality in hearing loss research.

## 3 Results

### 3.1 Bibliometric analysis

#### 3.1.1 Annual publication output and citation trends

**FIGURE 2:**
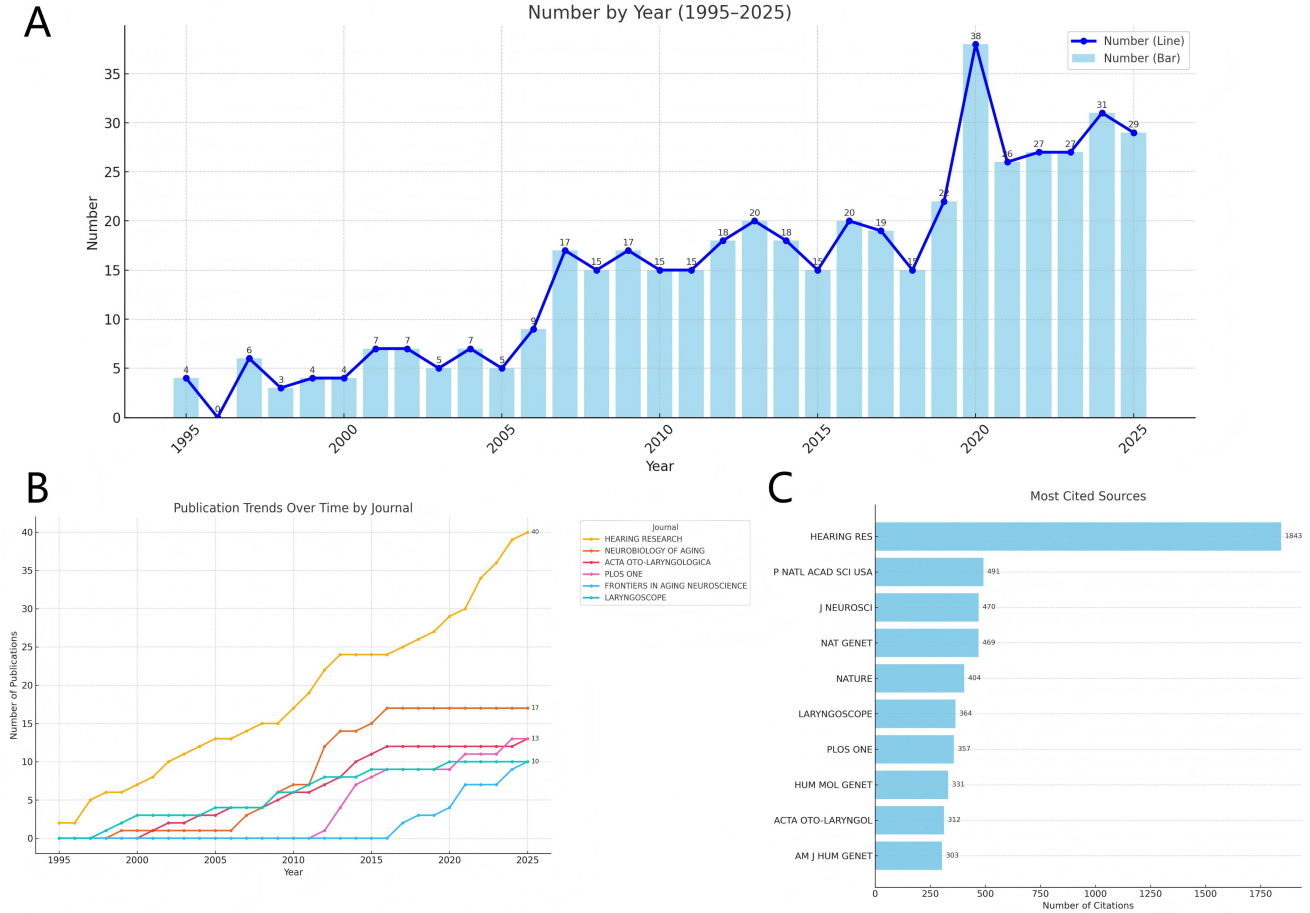
Trend in core publications. **(A)** Annual trends in publications. **(B)** Publication trends over time by journal. **(C)** Most cited sources.

Over the past three decades, a total of 2,483 authors has contributed to 465 publications across 190 journals in this field. Each article on average involved 8.06 co-authors, with each author contributed to approximately 2.45 publications. The overall research output has demonstrated a consistent upward trajectory, with an average annual growth rate of 6.83% (Figure 2A).

According to Bradford’s Law (Vickery, 1948), core journals were identified based on the distribution ratio of *1 : a : a^2^* (*a* is approximately 5), delineating core, related, and peripheral zones. Using this approach, we identified 13 core journals, as listed in Supplementary Table 3. As shown in Figure 2B, we analysed the publication trends of the top six journals by volume, which together account for 22.2% of total publications. Hearing Research emerged as the most prominent journal in both publication volume and growth trajectory. This dominance is further underscored in Figure 2C, where Hearing Research also leads in total citation count, with 1,843 citations, highlighting its central influence in the field.

#### 3.1.2 Authorship contribution and collaboration network analysis

**FIGURE 3:**
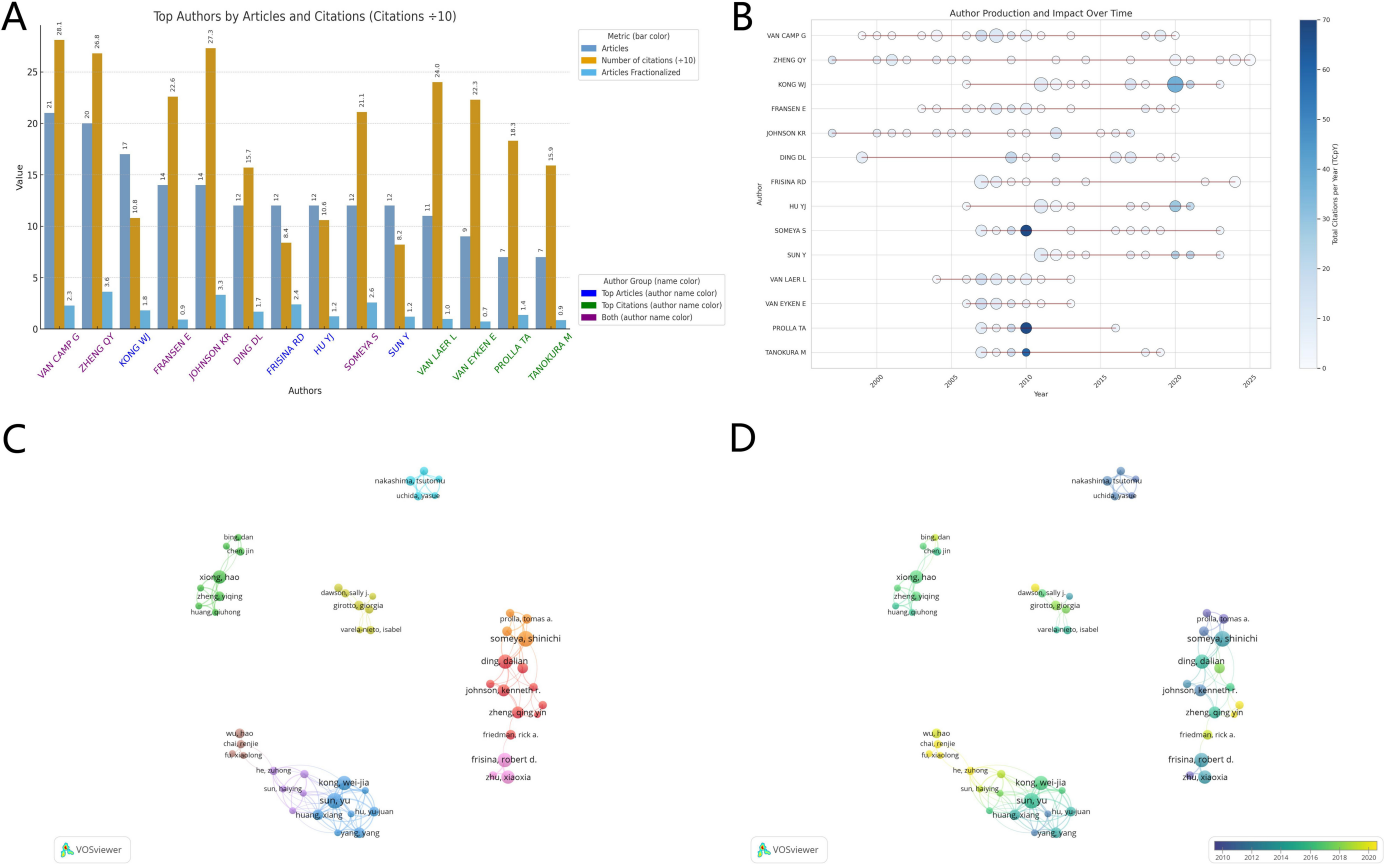
Author visualisation. **(A)** Top authors by number of articles and citations. **(B)** Author production trends. **(C)** Research partnerships that follow the topics and **(D)** the time.

According to the implications of Lotka’s Law (Price, 1965), among the 2,483 authors, 140 highly productive authors (5.64%) have published four or more articles within this field. However, author contribution cannot be accurately assessed by publication count alone. To provide a more nuanced evaluation, we also considered total citation frequency and average contribution share (Figure 3A). To avoid potential overestimation due to honorary or nominal authorship, we adopted the Articles Fractionalized (AF) metric as a more equitable measure of author contribution (Petroianu, 2012). Under this model, if a publication lists n authors, each is assigned a contribution value of 1/n, and an author’s total AF score is calculated by summing their fractional contributions across all publications, as defined by the AF model formula:


$$\mathrm{Frac}\,\mathrm{Freq}\,\left({\mathrm{AU}}_{j}\right)=\underset{h\in {\mathrm{AU}}_{j}}{\sum }\frac{1}{n.\mathrm{of}\,\mathrm{Co}\,\mathrm{Authors}\,\left(h\right)}$$

- AU_*j*_, is the set of documents co-authored by the author j
- H, is a document included in AU_*j*_

We identified six authors with substantial contributions to the field, each appearing in both the top 10 rankings for total publication count and overall citation frequency. Notably, Van Camp G ranked first in both total publications (*n* = 21) and total citations (*n* = 281). However, his Articles Fractionalized (AF) score (AF = 2.3) was lower than that of Zheng QY (AF = 3.6), who ranked second in publication count, and Johnson KR (AF = 3.3), who ranked second in total citations.

We visualised the annual publication output and citation frequency for these 12 prolific and highly cited authors (Figure 3B). In this plot, circle size represents the number of publications per year, while colour intensity indicates annual citation frequency. The earliest contributors identified in this field were Zheng QY and Johnson KR, both of whom began publishing in 1997 and have maintained consistent output. Over half of the prolific authors entered the field after 2005, and a notable surge in citation frequency occurred around 2010, marking a period of accelerated impact in the field.

To exemplify patterns of collaboration, an author co-authorship network was constructed using VOSviewer (Figures 3C, D). The visualisations show that most collaborations are nationally clustered, with authors predominantly collaborating within their own countries. The overall research network remains relatively fragmented. In Figure 3D, node colour reflects the year in which each researcher joined the collaboration network, with lighter shades indicating more recent entry. For example, we identified Wu Hao and Chai Renjie as authors who entered the collaborative landscape around 2020.

#### 3.1.3 Country and institutional contributions

**FIGURE 4:**
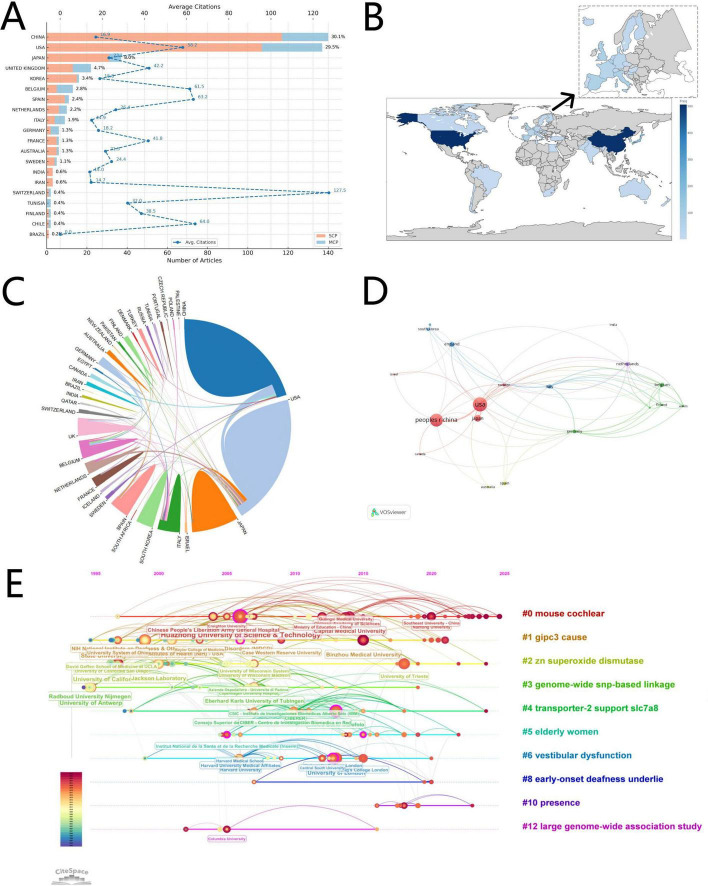
Countries and institutions visualisation. **(A)** Articles by country of corresponding author (with citations). **(B)** World map of publications distributed in various countries/regions. **(C)** Chord diagram of international collaboration. **(D)** International research cooperation network. **(E)** Institutional collaborative networks and timeline distribution clusters.

Authors contributing to this field were affiliated with 610 institutions across 34 countries. Based on the number of publications by corresponding authors, we identified the top 20 contributing countries, as shown in Figure 4A. The results showed that China (*n* = 140, 30.1%) and the United States (*n* = 137, 29.5%) published the most papers, far surpassing other countries. However, when considering average citations per article shown by the dotted line, a distinct contrast emerges. Although China ranks first in the number of publications in this field, its average citation frequency is only 16.9 times, significantly lower than the 58.2 times of the United States. This gap reflects that China still has considerable room for improvement in improving research quality and international influence. It is worth noting that Switzerland with relatively few publications, has an average citation count of 127.5, its contributions are highly influential, reflecting strong academic value and global recognition.

The relative proportions of Single Country Publications (SCP) and Multiple Country Publications (MCP) reveal a mainly domestic collaboration pattern across most countries, consistent with the author collaboration network depicted in Figure 3C. A geographic distribution map (Figure 4B) illustrates national publication frequencies, with darker shading representing higher output. International collaboration networks were visualised using Scimago Graphica and VOSviewer (Figures 4C, D), showing that while most collaborations remained local, few international partnerships between China and the United States were also observed, followed by collaborations involving East Asian countries (e.g., Japan, South Korea) and European nations (e.g., the United Kingdom, the Netherlands).

Secondly, this study analysed the publishing institutions in the field. At the institutional level, the top five most productive affiliations were Huazhong University of Science and Technology (*n* = 57), University of California System (*n* = 50), University of Antwerp (*n* = 40), State University System of Florida (*n* = 38), and Shanghai Jiao Tong University (*n* = 31). Using CiteSpace, we constructed a thematic timeline network (topics #0 to #12) to visualise institutional collaborations across specific research clusters (Figure 4E). For example, Huazhong University of Science and Technology and Capital Medical University collaborated under the #0 cluster (“mouse cochlear”), whereas University of Antwerp and Radboud University showed close collaboration in the #3 cluster (“genome-wide SNP-based linkage”).

#### 3.1.4 Publication theme analysis

**FIGURE 5:**
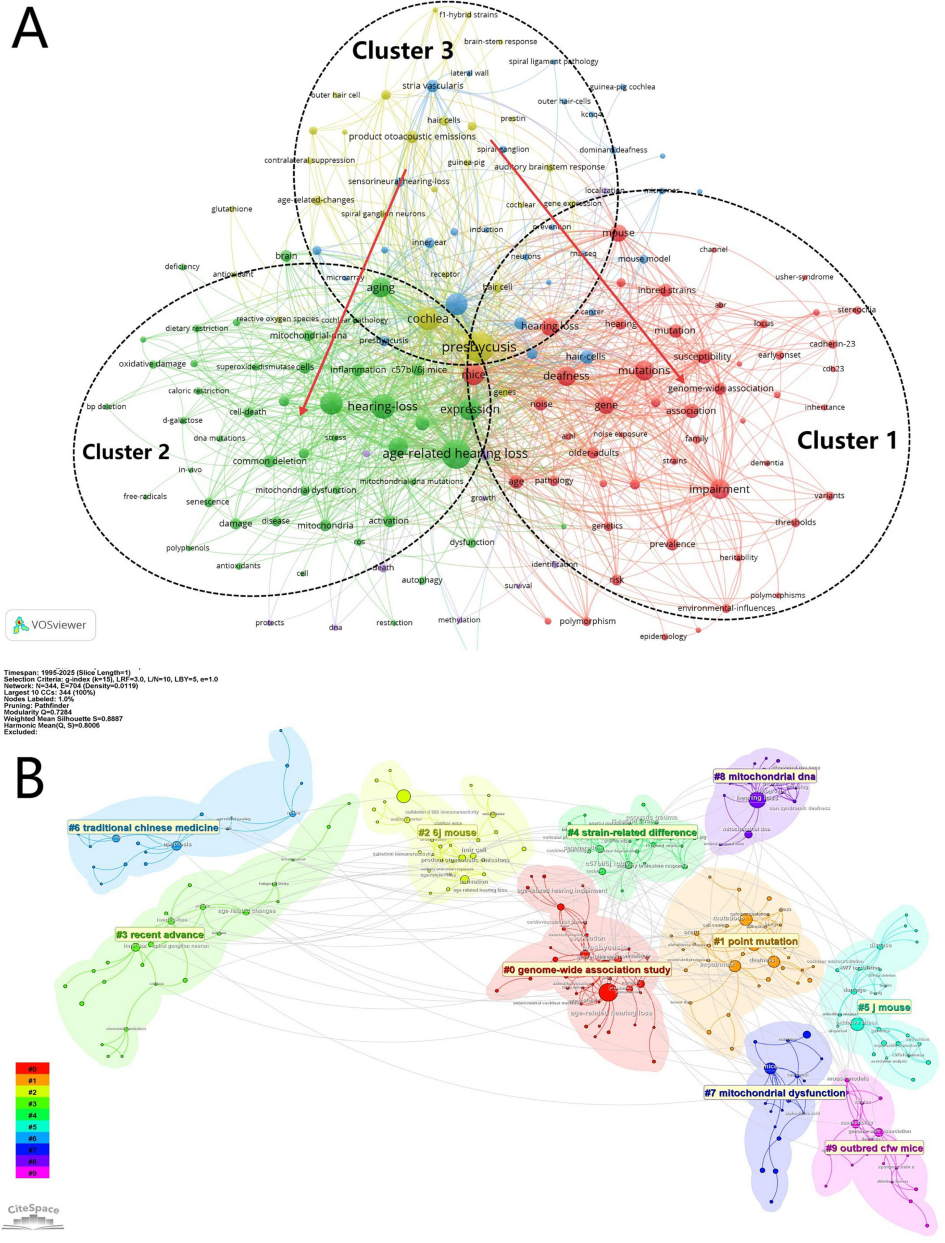
Topics and hotspots visualisation. **(A)** Keyword co-occurrence network. **(B)** Keyword cluster topic analysis.

To analyse thematic developments in the literature, we conducted keyword co-occurrence and cluster analysis using VOSviewer and CiteSpace. In VOSviewer, high-frequency terms from titles and abstracts were used to generate a keyword co-occurrence network, visualised in Figure 5A, which revealed three major thematic clusters, each indicated by a different node colour. Together with Supplementary Figure 1, we observed a clear thematic shift: research is gradually moving away from Cluster 3 focusing on inner ear anatomical structures such as spiral ganglion neurons and stria vascularis, toward Clusters 1 and 2. Cluster 1 focuses on genetic research related to ARHL, with terms like “mutation(s),” “genome-wide association,” and “polymorphism(s),” whereas Cluster 2 focuses cellular and molecular mechanisms, including “mitochondrial dysfunction,” “oxidative damage,” and “inflammation.”

Further thematic refinement was achieved through log-likelihood ratio (LLR) clustering in CiteSpace, shown in Figure 5B. The clustering results were evaluated using two indices: the clustering modularity value Q and the clustering contour index S (average contour). A Q value of 0.7284 ( > 0.5) indicates a well-defined cluster structure, while an S value of 0.8887 ( > 0.7) suggests high internal consistency and strong separation between clusters, indicating robust and credible clustering outcomes (Brandes et al., 2008). The clustering results shown in the figure reflect the current subfield-specific research hotspots. Six major thematic subfields were identified after merging overlapping clusters: #0: Genome-Wide Association Study, #1Point Mutation, #2/5/9 Mouse Model, #4 Strain-Related Difference, #6 Traditional Chinese Medicine, and #7/8 Mitochondrial-Related Mechanisms. These clusters represent the current research hotspots and key directions in the investigation of genetic factors associated with ARHL.

### 3.2 Functional categorisation of candidate gene research

**FIGURE 6:**
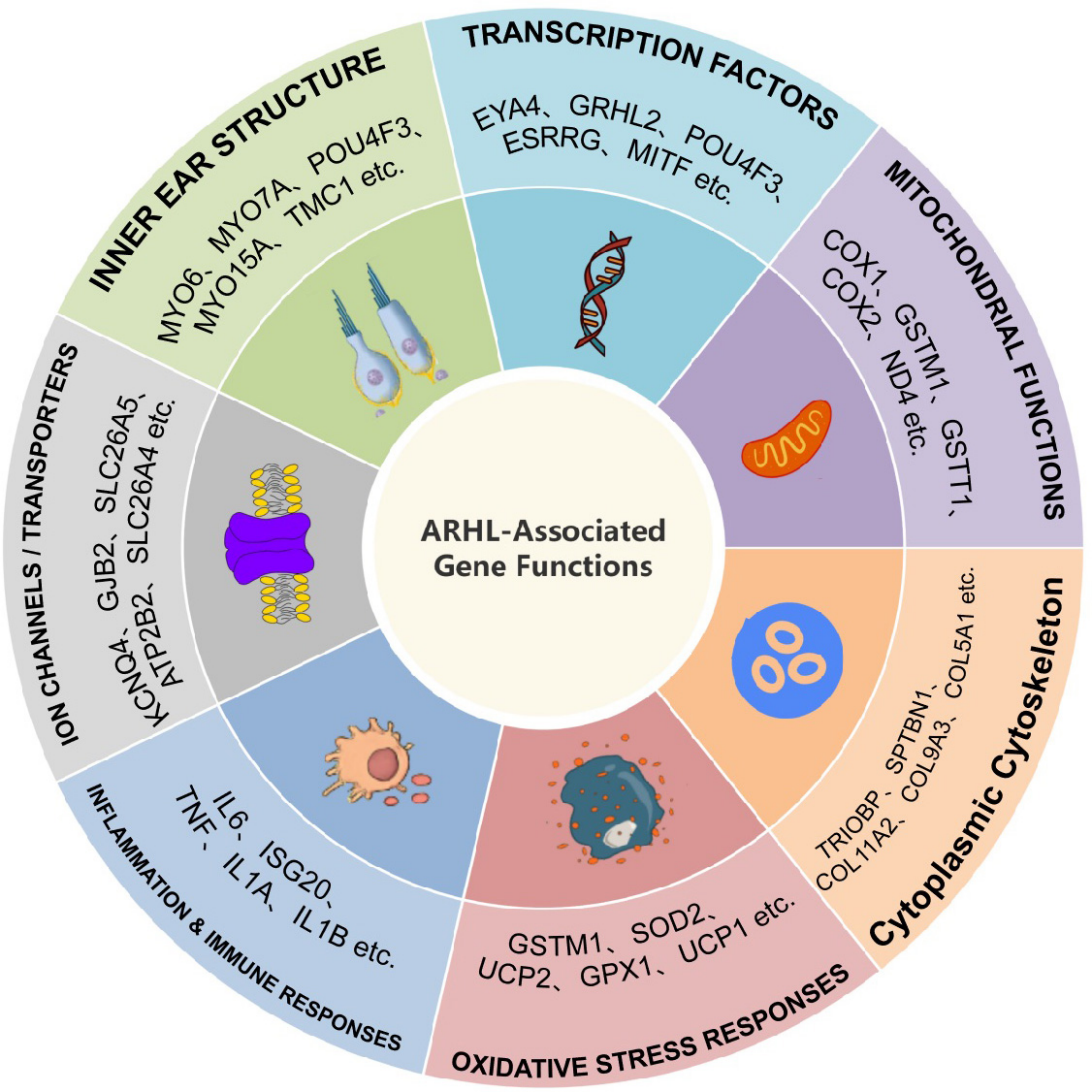
Functional categorisation of ARHL-associated genes.

In this study, 69.42% (*n* = 252) of the included articles were studies based on animal models, collectively reporting 365 candidate genes or genetic factors. Human studies accounted for 28.65% (*n* = 104), with a total of 221 candidate genes identified. Additionally, 1.93% (*n* = 7) of the studies employed a hybrid design incorporating both animal models and human samples. The sample regional distribution methods and methods of the included studies are presented in Supplementary Figure 2. The reported genes were categorised into seven major functional groups, including “Inner Ear Structure,” “Ion Channels/Transporters,” and “Transcription Factors,” among others (see Figure 6).

#### 3.2.1 Animal studies

**FIGURE 7:**
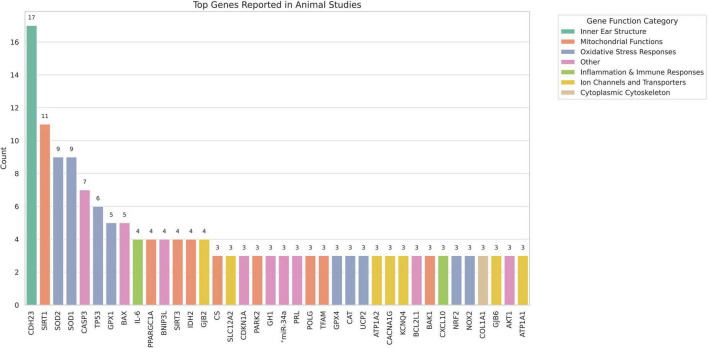
Top genes reported in animal studies.

This study systematically reviewed and analysed a total of 252 animal experimental studies related to ARHL, from which 365 genetic regulatory factors were extracted, including genes and various non-coding RNA molecules. To clarify the primary research foci within the current literature, we first performed a statistical analysis of the genetic regulatory factors that appeared three or more times across the included studies with visualisation (see Figure 7).

Among the 38 high-frequency genes identified, *Cdh23* was cited most frequently, appearing in 17 studies, followed by *Sirt1* (*n* = 11), *Sod2* and *Sod1* (*n* = 9 each), and *Casp3* (*n* = 7). Functional categorisation revealed that oxidative stress-related genes (e.g., *Sod2*, *Sod1*, *Gpx1*, *Cat*, and *Gpx4 etc.*,) constitute a substantial portion of the most frequently reported genes. In addition, mitochondrial function-related genes (e.g., *Sirt1*, *Sirt3*, *Tfam*, and *Polg etc.)* were also repeatedly highlighted. Inflammatory mediators (e.g., *Il-6*, *Il-1*β) and genes related to ion transport (e.g., *Kcnq4*, *Gjb2*, *Atp1a2 etc.*,) were likewise represented. In addition, most of the genes in the “Other” category belong to genetic factors related to cell apoptosis and anti-apoptosis. The frequent recurrence of these genes illustrates the current research focus in ARHL animal studies on mechanisms such as oxidative stress, mitochondrial dysfunction, and inflammation.

#### 3.2.2 Human studies

In the total of 111 studies investigating the genetic basis of ARHL in human subjects, 210 potential gene loci were extracted. All identified loci were compiled, and genes reported five or more times were summarised alongside their corresponding functional categories (see Table 1). Although genes such as *EYA4, GRM7, NAT2, KCNQ4, CDH23*, and *GSTM1* differ in their functional categories, they all play an important role in the pathogenesis of ARHL.

**TABLE 1:** High-frequency genes (≥ 5) identified in ARHL studies and their functions.

Genes	Functions	Frequencies
EYA4	Transcription factors	7
GRM7	Neurotransmitter signaling	7
NAT2	Oxidative stress responses	7
KCNQ4	Ion channels / transporters	6
GRHL2	Transcription factors	6
MYO6	Inner ear structure	5
CDH23	Inner ear structure	5
GSTM1	Oxidative stress responses	5

Studies based on European populations accounted for over 35% (36.76%) of the total human studies and demonstrated greater depth, width, and methodological diversity compared to those focusing on other ethnic groups (see Supplementary Figure 1). Therefore, we compiled the top ten most frequently reported genes within European cohorts, as well as the top five genes reported among other populations, along with their associated functional categories (see Supplementary Table 4).

### 3.3 Cross-ethnic analysis of candidate genes

**FIGURE 8:**
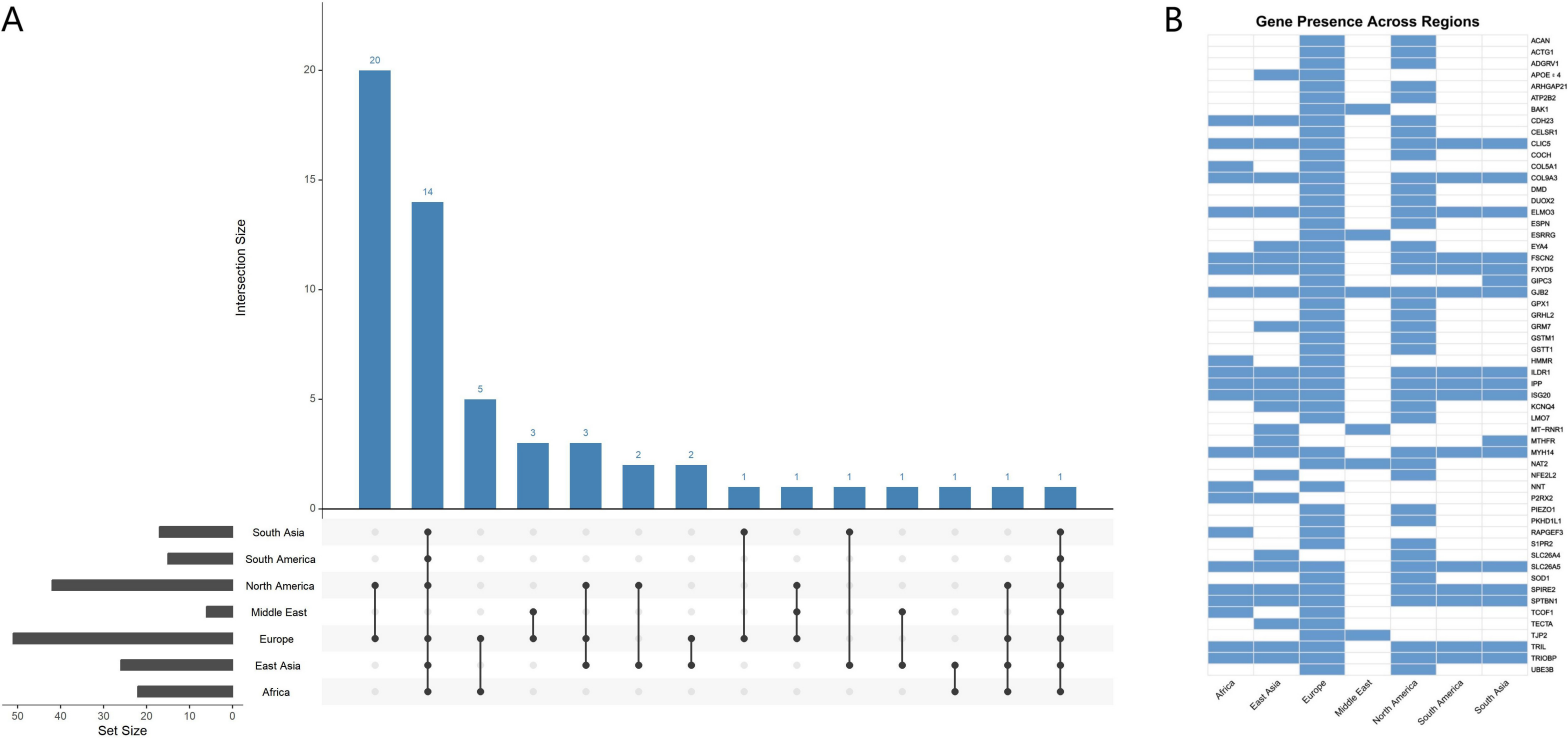
Cross-ethnic gene research network in ARHL studies. **(A)** UpSet plot illustrating the intersection of ARHL-related genes across ethnic/geographic groups. **(B)** Heatmap displaying candidate gene distribution.

This study builds a cross-ethnic gene network by integrating 56 out of the 210 candidate genes that have been reported across diverse ethnic populations. Figure 8A illustrates the intersectional distribution of these genes across studies from different ethnic or geographic regions. Of the 56 shared genes, 20 were reported in both European and North American populations, representing the largest overlap set. The second largest intersection consists of a group of 14 genes reported across studies from Europe, North America, South America, East Asia, South Asia, and Africa. Moreover, several smaller overlapping clusters were observed, involving combinations of two or more regions. For example, Europe and Africa (5 genes), Europe and the Middle East (3 genes), and Europe, North America, and East Asia (3 genes). It is worth to mention that European studies reported the greatest number of genes, followed by those from North America and East Asia, highlighting their substantial contributions in discovering ethnicity-specific gene. These findings indicate a degree of gene sharing across geographic regions, while also highlighting distinct patterns of regional specificity. To further elaborate on the intersection patterns presented in Figures 8A, B visualises the distribution of specific candidate genes studied in different geographic populations. Displayed in a matrix format, this figure provides an intuitive overview of the population-specific reporting patterns for each shared gene. It is found that certain genes, such as *CDH23*, *ILDR1*, and *SLC26A5*, have been repeatedly reported across multiple ethnic groups, demonstrating strong cross-ethnic consistency. These genes are therefore considered high-priority candidates in ARHL research. Collectively, these findings suggest that certain genes may have broad relevance across diverse genetic backgrounds, making them promising targets for future multi-ethnic integrative analyses.

## 4 Discussion

### 4.1 Current research and trends

Our findings indicate a steadily increasing research interest in the genetic factors underlying ARHL. The sustained average annual growth rate of 6.83% in publications not only signifies heightened scholarly focus but also points to considerable opportunities for further investigation. This trend was particularly pronounced from 2019 to 2020, a period that saw an exceptional 72.72% spike in research output. Publications are primarily concentrated in journals dedicated to two main disciplines: hearing science and geriatrics. Within this scope, the journal *Hearing Research* demonstrates notable prominence. It has contributed the highest number of publications (8.60%, 40/465) and credited to the highest number of citations (*n* = 1,843), reflecting its central role and significant impact in ARHL genetics.

Research findings in this domain have appeared in top-tier science journals, including *Cell* and *Nature*. However, no related studies have been published in the four leading general medical journals: *The New England Journal of Medicine (NEJM)*, The *British Medical Journal (BMJ), The Journal of the American Medical Association (JAMA)*, and *The Lancet*. This distribution suggested that while the field has achieved significant advances in fundamental and interdisciplinary research, clinically oriented translational studies remained relatively limited compared to basic science research. Consequently, a future imperative is to bridge this translational gap by facilitating the effective transfer of basic science discoveries into clinical applications. Such efforts are critical to realising the clinical value of genetics in the prevention, diagnosis, and treatment of ARHL.

Currently, the most prolific authors in the field of ARHL genetics are predominantly based in China and the United States, with both countries maintaining a high level of research activity in this domain. However, it is worth noting that Chinese scholars entered this field relatively recently, with most of their contributions published after 2020. In contrast, American scholars had already conducted research on the genetic mechanism of ARHL as early as around 2010, building a more extensive foundation of basic research and scholarly influence. This temporal disparity reflects, to some extent, the gap between the two nations in research accumulation and international influence.

At the national level, China and the United States are currently the two leading contributors, accounting for 30.1% (140/465) and 29.5% (137/465) of publications in the field, respectively. Despite their strong publication output, existing research collaboration networks remain largely confined within domestic institutions, with relatively limited cross-national cooperation. This pattern may be influenced by several factors. First, ARHL genetic research often relied on population-specific samples and linguistic contexts, making data collection inherently region-dependent. Second, differences in ethical approval procedures, sample-sharing mechanisms, and data privacy regulations across countries added further complexity to international collaboration. In addition, most funding in this field was derived from national or regional programmes, which may have restricted the depth and breadth of cross-national cooperation. These factors collectively contributed to the current fragmentation of global research networks, potentially limiting resource sharing, methodological exchange, and the broader dissemination of findings in ARHL genetics. On the other hand, research from Africa, the Middle East, and Latin America has been markedly deficient. This gap is rooted not only in a lack of interest or researchers, but also due to a series of structural obstacles. Firstly, the capacity for large-scale genomic research was possibly hindered by a lack of advanced sequencing platforms and specialised bioinformatics talent. Secondly, data availability and comparability may have been constrained by a scarcity of high-quality longitudinal cohorts and robust databases. Thirdly, at a foundational level, sample collection may have been hampered by insufficient primary healthcare infrastructure, while limited national health expenditures may have resulted in inadequate funding for studies on aging and hearing. Compounding these issues are underdeveloped regulatory and ethical frameworks for genetic research and a lack of established protocols for international data sharing. Together, these barriers were responsible for the skewed geographical distribution of research in this field.

Looking forward, strengthening research synergies between China, the United States, and other nations would be essential. Building multinational research alliances, sharing large-scale genetic databases, and pooling multicentre clinical sample resources will help broaden the scope and depth of investigation to more comprehensively reveal the genetic mechanisms of ARHL and accelerate the pace of translation from basic research to clinical practice.

### 4.2 Shifting of research hotspots

Current research priorities in ARHL have progressively shifted from traditional investigations of inner ear anatomy and physiological structures toward a deeper exploration of its molecular and genetic mechanisms. Earlier studies focused on histopathological changes within the cochlea, categorising ARHL into the sensory type (loss of outer hair cells), the neural type (degeneration of auditory nerve), and the metabolic type (degeneration of the spiral ganglion and atrophy of the stria vascularis) (Yang et al., 2023). These degenerative anatomical changes were long considered the primary cause of age-related hearing decline. With advances in molecular biology and genetics, ARHL research focus has gradually shifted from macroscopic histopathology to microscopic molecular mechanisms. Increasing genetic evidence has depict the critical role of inherited susceptibility in the pathogenesis of ARHL. For instance, Bowl and Dawson (Bowl and Dawson, 2019), through twin and family-based studies, estimated the heritability of ARHL to be between 35% and 55%, suggesting the significance of genetic predisposition in its development and progression.

Mouse models also facilitated the revelation of the genetic mechanisms of ARHL. In aged, inbred strains such as C57BL/6J, *Ahl* locus is identified on chromosome 10 and found to be associated with progressive hearing decline in mice (Keithley et al., 2004). Subsequent studies have shown that *Ahl* corresponds to the gene Cadherin 23 (*CDH23*), which encodes a hair cell adhesion protein, with its mutations leading to progressive hearing loss in mice (Johnson et al., 2008; Liu et al., 2012). Echoing this, *CDH23* mutations have also been detected in human ARHL patients (Zheng et al., 2005). The discovery of the *Ahl* locus marked a shift in ARHL research from anatomy and physiological structure of the inner ear to molecular genetic mechanisms, suggesting that inner ear degeneration may stem from specific genetic defects or inherited susceptibilities. Since then, candidate gene association studies (GAS) and GWAS have identified numerous ARHL-related susceptibility loci, providing new insights into molecular mechanisms and the development of potential therapeutic targets. Representative susceptibility loci identified to date include *GRM7* (Friedman et al., 2009), *IQGAP2* (Van Laer et al., 2010), *ESRRG* (Nolan et al., 2013), *ILDR1*, *TRIOBP*, and *EYA4* (Hoffmann et al., 2016), among others. Collectively, these loci highlighted the complex and polygenic nature of ARHL, reflecting the multifactorial genetic architecture underlying age-related auditory decline.

Emergence of research hotspots such as mitochondrial dysfunction, oxidative stress, and inflammation have shaped new research directions toward deeper mechanistic exploration. For example, the mtDNA 4977 deletion was considered a hallmark of mitochondrial impairment in ARHL. With the advancement of ageing biology, researchers have increasingly incorporated theories of oxidative stress and mitochondrial dysfunction into investigations of auditory ageing. Concurrently, inflammation, recognised as a key driver of the ageing process, has also gained growing attention in ARHL research. These gene-mediated pathogenic mechanisms will be examined in detail in the following sections of this study.

Among the thematic clusters identified in this study, one particularly novel research direction emerged: Cluster #6 Traditional Chinese Medicine (TCM) (see Figure 5B), as an example of increasing attention directed toward effective strategies for delaying or intervening in auditory ageing beyond conventional biomedical approaches such as pharmacological treatment, hearing aids, and cochlear implants. According to traditional Chinese medical theory, age-related hearing decline was often attributed to “kidney qi deficiency,**”** a concept that has received preliminary support from contemporary studies (Thodi et al., 2006). A wide range of supplementary herbal formulations have been long applied in clinical auditory health management. For instance, the classical formula *Er Long Zuo Ci Wan* (a pill treating tinnitus and hearing loss), has been historically documented to treat ARHL in TCM compendiums. In recent years, TCM practices have been increasingly examined and translated through modern scientific methodologies. Research were focused in isolating active compounds from herbal formulas and exploring their underlying mechanisms of action using cellular and animal models, thereby bridging traditional knowledge with molecular-level validation.

A growing body of experimental evidence suggested that TCM and their active compounds exert a wide range of biological effects, such as antioxidant, anti-inflammatory, microcirculatory improvement, and anti-apoptotic activities, all of which directly target key pathogenic processes implicated in genetically mediated ARHL (Hu et al., 2023). For instance, *Celastrol*, a principal bioactive component derived from *Tripterygium wilfordii Hook. f.* (Lei Gong Teng), has been shown in animal models to significantly promote the viability and proliferation of inner ear stem cells, induce their cell differentiation into neuron-like cells, and enhance neuro-excitability and electrophysiological activity. These effects were potentially mediated by the upregulation of *Atoh1*, a critical transcription factor governing inner ear sensory cell differentiation (Han et al., 2018). Similarly, *Sesamin*, extracted from *Sesamum indicum L.* (black sesame), has been reported to upregulate the expression and activity of the hearing-related gene *Tecta*, thereby exerting protection against auditory cell ageing (Kim et al., 2020).

### 4.3 Functional categorisation of ARHL-related genes

To reveal the complex genetic architecture of ARHL, candidate genes were systematically stratified into seven categories based on their primary pathophysiological mechanisms (Figure 6). Building upon this framework, the following section provides a detailed discussion of representative genes within each category, focusing on their molecular functions, evidence in animal models and human studies, pathway involvement, and their contributions to ARHL.

#### 4.3.1 Inner ear structure genes

The integrity and function of the inner ear are critical for normal hearing. Multiple genes involved in the regulation of inner ear architecture and intercellular connectivity have been implicated in susceptibility to ARHL. Variants in these genes may contribute to age-dependent degeneration of hair cells, supporting cells, or the basilar membrane, ultimately leading to progressive auditory decline.

*CDH23* gene encodes Cadherin-23, an adhesion protein constituting the tip links of stereocilia. Early studies in mouse models revealed that a single nucleotide variant in *CDH23* (753A > G) significantly accelerates the progression of ARHL. Mouse carrying the 753A allele exhibit rapid age-related auditory decline, while those with the wild-type 753G allele demonstrate partial protection, particularly against high-frequency hearing loss (Johnson et al., 2017). The widely used C57BL/6 strain carries the *CDH23^ahl* variant (753A), and targeted substitution with the protective 753G allele leads to significantly improved auditory thresholds at 15 months of age (Johnson et al., 2017). In humans, *CDH23* is a well-established gene involved in congenital hearing loss. Recent studies have also implicated *CDH23* in ARHL, with high methylation levels of this gene associated with elevated ARHL risk among older adults (Bouzid et al., 2018). Structural biology investigations further support the pathogenicity of *CDH23* dysfunction. Mutations such as *S47P* have been shown to compromise the stability and folding efficiency of Cadherin-23, impairing its adhesive role in tip link formation. This conformational disruption not only interferes with mechanotransduction but may also accelerate age-related degeneration of hair cells (Garg et al., 2021).

*MYO6* encodes myosin VI, an actin-based motor protein located in the cytoplasm of cochlear hair cells within the organ of Corti, anchoring the structural integrity of stereocilia (Self et al., 1999). Pathogenic variants in *MYO6* have been identified as causative factors in both autosomal dominant DFNA22 and autosomal recessive DFNB37 forms of hereditary hearing loss (Ahmed et al., 2003; Melchionda et al., 2001). Subsequent pedigree study have implicated a missense mutation (*c.3610C > T; p.R1204W*) as potential contribution to the onset of ARHL (Oonk et al., 2013).

*TECTA* encodes α-tectorin, a structural protein found in the extracellular matrix of the inner ear’s tectorial membrane. Recent exome-wide association studies of individuals with ARHL in the UK Biobank have shown that rare variants in the *TECTA* gene are significantly enriched in patients with ARHL, with this finding validated in a large cohort study of the Japanese population (Cornejo-Sanchez et al., 2023; Yasukawa et al., 2019).

#### 4.3.2 Transcription factors

Transcription factors are essential in regulating inner ear development, stress response, and cell survival (Li et al., 2016). With aging, the expression and activity of various transcription factors changes, thereby disrupting downstream gene regulation and contributing to the ageing of the auditory system.

*EYA4* belongs to the vertebrate *Eya* gene family and functions as a transcriptional activator (Wayne et al., 2001). Recent genetic analyses in older populations have revealed a significant enrichment of rare *EYA4* variants among individuals with ARHL, suggesting its possible involvement in the genetic architecture of common, late-onset hearing impairment (Cornejo-Sanchez et al., 2023). Earlier positional cloning studies also identified truncating mutations in *EYA4* across multiple families associated with progressive hearing loss beginning in adulthood (Wayne et al., 2001).

*GRHL2*, also known as *TFCP2L3*, encodes an epithelium-specific transcription factor. Mutations in *GRHL2* have been identified to cause DFNA28 progressive hearing loss in humans (Vona et al., 2013). Large-scale association studies in multiple European populations have revealed a significant correlation between *GRHL2* and ARHL, particularly involving multiple SNPs located within intron 1. Given that these variants reside in non-coding regions, researchers hypothesised that they may alter *GRHL2* expression levels instead of protein structure, thus affecting the survival and function of cochlear epithelial cells, ultimately contributing to the auditory ageing process (Van Laer et al., 2008).

*FOXO3* (*Forkhead box O3*) and *POU4F3* have also been implicated in cochlear hair cell differentiation and survival. Variants or loss of function in these genes may reduce the ability of hair cells to withstand age-related damage, including noise and metabolic stress, leading to higher susceptibility to degeneration or apoptosis in hair cells (Gilels et al., 2017; Singh et al., 2024).

#### 4.3.3 Mitochondrial functions and oxidative stress-related genes

Reactive oxygen species (ROS) are signalling molecules for cellular proliferation and survival. Yet, their excessive production and accumulation trigger oxidative stress (OS), which accelerates the degeneration of cochlear hair cells and auditory neurons (Ray et al., 2012). With ageing, ROS levels in the inner ear tissues increase, while antioxidant defence decline. This disequilibrium is widely regarded as a central pathogenic mechanism of ARHL. Seidman et al. (2002). further demonstrated that ROS accumulation in the cochlea can induce mutations in the mitochondrial genome, leading to mtDNA damage that disrupts cellular energy metabolism and compromises cell viability. In support of this, Markaryan et al. (2009) observed in human temporal bones, a significantly reduced expression of mitochondrial enzymes involved in cochlear tissues of elderly individuals with ARHL, indicating impaired mitochondrial metabolic function. Importantly, a vicious cycle may exist between ROS accumulation and mitochondrial dysfunction: ROS accumulation induces mitochondrial damage, and in turn, compromised mitochondria further elevate ROS production. This feedback loop ultimately accelerates the degeneration of cochlear hair cells and neurons, driving the progression of ARHL.

*COX1* is a subunit of respiratory chain complex IV encoded by the mitochondrial genome, it plays a critical role in the high energy metabolism of cochlear hair cells (Dennerlein et al., 2023). Animal studies have demonstrated that significant downregulation of *COX1* is positively associated with mitochondrial morphological degeneration, impairment of auditory hair cells, and the severity of hearing loss (Lyu et al., 2020). Similarly, in human temporal bones, reduced expression of cytochrome c oxidase encoded by *COX1* has been observed in the spiral ganglion neurons of the cochlea in elderly individuals, possibly linked with accumulated mtDNA mutations (Keithley et al., 2001).

*SIRT3* is a mitochondrial NAD^+^-dependent deacetylase. It modulates the activity of antioxidant and metabolic enzymes within mitochondria and has been identified as a key protective factor against auditory ageing. A pivotal study by Someya et al. (2010), published in *Cell*, showed that caloric restriction (CR) significantly delayed hearing decline in wild-type mice, whereas *Sirt3* knockout mice exhibited progressive hearing loss despite CR intervention. Mechanistically, CR activates the *SIRT3*-mediated deacetylation and upregulation of mitochondrial antioxidant enzyme *Mn-SOD* (*SOD2*), enhancing glutathione-based antioxidant defence and thereby mitigating ROS-induced damage to cochlear hair cells (Zeng et al., 2014). Collectively, these findings suggest that the CR-*SIRT3*-*SOD2* pathway may be one of the core mechanisms regulating auditory system aging.

Mutations in the mitochondrial gene *MT-RNR1* are associated with ARHL. The A1555G mutation in 12S rRNA not only makes carriers highly susceptible to aminoglycoside-induced ototoxicity, but also often causes sensorineural hearing loss in older adults even in the absence of antibiotic exposure (Teraoka et al., 2024). This mutation increases mitochondrial susceptibility to damage by ROS and noise. Owing to the maternal inheritance pattern and high copy number characteristics of mtDNA, the A1555G mutation is often observed in multiple elderly members within affected families, making it a typical example of mitochondrial-related ARHL (Mutai et al., 2017).

*SOD1* and *SOD2* encode antioxidant enzymes responsible for scavenging superoxide anions (O_2_^–^), with *SOD1* localised in the cytoplasm and *SOD2* in mitochondria. Studies have demonstrated that their expression levels decline progressively with age, leading to reduced cochlear antioxidant capacity and accumulation of ROS, ultimately triggering hair cell degeneration (McFadden et al., 1999; Yang et al., 2023). *SOD1*-knockout mice exhibited pronounced hearing loss and outer hair cell degeneration, whereas *SOD2* heterozygous-deficient mice showed increased auditory system vulnerability under oxidative stress.

#### 4.3.4 Inflammation and immune response genes

Emerging evidence suggests that inflammation and immune responses within the inner ear play a significant role in the onset and progression ARHL (Oonk et al., 2013). Aged individuals frequently exhibit a state of low-grade chronic inflammation, termed *inflammaging*, which also manifests in the cochlea as elevated levels of inflammatory mediators and increased immune cell infiltration (Gu et al., 2025; Qiu et al., 2024). Recent studies have indicated that anti-inflammatory interventions may partially attenuate the progression of auditory aging (Zhang et al., 2024).

*ISG20* encodes a 3’–5’ exonuclease that functions as an antiviral protein induced by the type I interferon pathway (Deymier et al., 2022). While initially investigated for its role in degrading viral RNA and mediating innate antiviral responses (Wu et al., 2019), recent genomic studies have suggested a potential association between *ISG20* and age-related auditory degeneration. A large-scale ARHL GWAS found that the *ISG20* gene locus was significantly associated with hearing loss in elderly people, but its specific mechanism is still unclear (Nagtegaal et al., 2019). Interestingly, other genes in the interferon pathway also showed differential expression, suggesting that chronic immune inflammation or antiviral responses in the inner ear may be involved in the process of auditory aging. It is hypothesised that *ISG20* may modulate the cochlear response to viral infections or inflammatory stimuli, thereby indirectly influencing hair cell or auditory neuron survival related to ARHL.

*IL-6* is a prototypical pro-inflammatory cytokine that plays a key role in the inflammatory response following inner ear injury. Experimental studies have shown that *IL-6* levels are significantly elevated in both the cochlea and serum of aged mice compared to younger controls (Lu et al., 2024). These inflammatory mediators can trigger harmful signalling pathways, such as NF-κB, which disrupt synaptic integrity and cellular function within the auditory system (Zhang et al., 2019). *IL-6* deficient mice exhibit attenuated age-related threshold shifts and reduced loss of cochlear ribbon synapses and spiral ganglion neurons, relative to wild-type controls during aging (Lu et al., 2024). These findings suggest that upregulation of *IL-6* reflects activation of cochlear immune-inflammatory responses. Persistent *IL-6* signalling may exacerbate the inflammatory microenvironment surrounding hair cells, thereby accelerating auditory neural circuit degeneration.

In addition to *IL-6*, other pro-inflammatory cytokines, such as tumour necrosis factor-alpha (*TNF*-α) and interleukin-1β (*IL-1*β), also demonstrate significant upregulation in the aging cochlea. Both animal models and elderly human studies have consistently reported elevated levels of these cytokines in cochlear tissue and systemic circulation (Lyu et al., 2020; Verschuur et al., 2012), outlining a broader inflammatory landscape implicated in ARHL.

#### 4.3.5 Ion channels and transporters

The normal function of the auditory system depends on a regulated ionic environment within the inner ear, maintained by various ion channels and transporters responsible for endolymph production, electrochemical gradients, and transmembrane ion flux. With aging, this ionic homeostasis becomes progressively dysregulated, often manifesting as altered electrolyte concentrations and endocochlear potential within the cochlear fluids.

*KCNQ4* encodes the voltage-gated potassium channel Kv7.4, which is mainly distributed on the basolateral membrane of the outer hair cells of the cochlea and is responsible for regulating the resting potential of the outer hair cells and the potassium recycling cycle (Kharkovets et al., 2006). In aged animal models with hearing loss, activation of Kv7.4 channels significantly preserved auditory threshold and OHC survival, suggesting its role as a key regulator of potassium efflux and membrane stability in aging cochlear cells (Peixoto Pinheiro et al., 2022). In human studies, Van Eyken et al. (2006) were among the first to establish a direct association between *KCNQ4* polymorphisms and ARHL through genetic analysis across two independent elderly Caucasian cohorts. They conducted a statistical analysis of 23 SNPs in the *KCNQ4* gene and found that multiple sites were significantly positively correlated with ARHL.

*SLC12A2* encodes the Na^+^-K^+^-2Cl^–^ symporter NKCC1, mainly located in the basilar membrane of the marginal cells of the stria vascularis as a key transporter involved in the endolymphatic potassium circulation (Bazard et al., 2021). NKCC1 maintains the inner ear ion microenvironment. Its expression decreases with age, leading to insufficient endolymphatic potassium ion supply and reduced cochlear potential (EP), which is one of the important mechanisms of ARHL. However, interestingly, studies have found that in double-transgenic mice with simultaneous knockout of NKCC1 and Na^+^/K^+^-ATPase α1 subunit, the progression of ARHL is actually delayed (Diaz et al., 2007), suggesting that there may be complex compensatory mechanisms between different ion transporters.

#### 4.3.6 Cytoplasmic cytoskeleton genes

The normal function of hair cells and supporting cells depends on a well-developed cytoskeletal structure. For example, the scaffold of stereocilia is composed of F-actin filaments and their cross-linking proteins. The cytoskeleton is also involved in maintaining synaptic structure and mechanical transduction mechanisms. With aging, the accumulation of microdamage to cytoskeletal components can lead to the degeneration of hair cell morphology and function (Wu and Liberman, 2022).

Variations in genes such as *TRIOBP* (TRIO binding protein), *SPTBN1* (beta-2 erythroid-free spectra protein), and *FSCN2* (Fascin-2) have been identified as loci associated with hearing aging in large-scale GWAS. The recurrent identification of SNPs within these genes suggests that alterations in cytoskeletal regulation may, to some extent, accelerate hearing deterioration in aging populations (Ivarsdottir et al., 2021; Nagtegaal et al., 2019).

#### 4.3.7 Common genetic pathways of ARHL and cognitive impairment

A substantial body of longitudinal research indicated a significant correlation between ARHL and cognitive impairment. The 2020 Lancet Commission identified midlife hearing loss as a major modifiable risk factor for dementia (Livingston et al., 2020). Two potential underlying mechanisms, for example, include the “cognitive load hypothesis,” which posited that hearing loss increases cognitive load and depletes resources available for other cognitive tasks (Wayne and Johnsrude, 2015), and the “common cause hypothesis,” which suggested that hearing loss and dementia share common neuropathological mechanisms (Shen et al., 2018). Furthermore, genetic and molecular studies reveal that ARHL and cognitive impairment may share overlapping susceptibility genes and pathological pathways.

To date, the Apolipoprotein E (APOE) ε4 allele has been widely recognised as one of the strongest genetic risk factors for Alzheimer’s disease (AD), with carriers facing a three- to four-fold increased risk of developing the disease (Smith and Ashford, 2017). Congruently, in our study, *APOE* ε4 was also identified as a high-frequency genetic locus associated with ARHL. Supporting this link, a Mendelian randomisation study by Abidin et al. (2021) confirmed that genetic liability for AD directly contributed to hearing loss, with the effect being predominantly driven by *APOE* ε4. This evidence suggested that *APOE* ε4 may be a key upstream factor connecting ARHL and AD. Its presence could accelerate auditory system aging through peripheral pathways (e.g., atherosclerosis leading to insufficient cochlear blood supply) while simultaneously promoting the decline of both auditory and cognitive functions through central pathways (e.g., facilitating amyloid-beta deposition and neuroinflammation in the brain) (Abidin et al., 2021; Han et al., 2024).

Additionally, the *GRM7* gene, which encodes the metabotropic glutamate receptor 7 (mGluR7), was proposed to influence both conditions. It primarily regulates glutamate release and synaptic plasticity at the presynaptic membrane of neurons. Synaptic plasticity is crucial for both auditory and cognitive functions. Variations in *GRM7* may reduce the efficiency of synaptic transmission in the auditory pathway and may concurrently diminish the plasticity reserve in other cognitive-related neural networks, thereby dually increasing susceptibility to both hearing loss and dementia (Chaumette et al., 2022; Friedman et al., 2009; Pérez-Palma et al., 2014).

Other ARHL susceptibility genes also exhibited crossover mechanisms with cognitive functions. For instance, *SLC6A4* and the serotonin pathway were thought to be involved in the regulation of brain and auditory health through mood and social interaction (Fratelli et al., 2020); serotonin (5-HT), which is related to *SLC6A4* function, may indirectly contribute to cognitive impairment while modulating psychosocial factors (Keesom and Hurley, 2020). Similarly, Brain-Derived Neurotrophic Factor (BDNF) is a critical protein responsible for central and peripheral neurons survival and supporting synaptic plasticity. The functional polymorphism of the BDNF gene (Val66Met) was suggested to be associated with cognitive performance and neurodegenerative disease risk (Chao et al., 2022), while reduced BDNF levels were also considered a key factor in age-related alterations of plasticity within the central auditory system (Rüttiger et al., 2007).

### 4.4 Significant findings in animal models

Although some genes appear infrequently in the literature, the mechanisms they implicate offer substantial research value. For example, *Glra1* is involved in inhibitory neurotransmission in the central auditory pathway. Studies in aged mouse model have shown that changes in *Glra1* expression in the dorsal cochlear nucleus of the brainstem correlates with reduced auditory nerve synchrony, suggesting that central inhibitory dysregulation may play a role in age-related auditory decline (Wang et al., 2009). *Coch* encodes the cochlear matrix protein cochlin, and *Cdh23* encodes the connexin Cadherin-23. These two genes usually do not show obvious phenotypes in wild-type animals, and hearing loss only occurs in specific mutant models (Jones et al., 2011; Newton et al., 2023). In addition, *Txnip*, *Idh3a*, and *Bmi1* represent mechanisms such as oxidative-inflammatory coupling, mitochondrial metabolic regulation, and stem cell homeostasis, respectively.

*Txnip* can amplify OS and activate inflammatory pathways by inhibiting thioredoxin (Trx). Studies have confirmed that it plays a key regulatory role in the ROS/NLRP3 pathway in auditory cell models (Ma et al., 2024). *Idh3a* is a key metabolic enzyme in the tricarboxylic acid (TCA) cycle. Although relatively few studies have been conducted on it, it plays an important role in regulating the NADH/NAD^+^ balance and is crucial for maintaining mitochondrial function (Yang et al., 2023). *BMI1* deficiency can lead to premature apoptosis of OHCs and ROS accumulation. Studies in mouse models have shown that *Bmi1* plays a protective role in maintaining hair cell homeostasis and is highly sensitive to oxidative damage (Chen et al., 2015). Overall, although these genes with low research frequency have not become hot topics, the mechanisms involved have enriched our understanding of the pathogenesis of ARHL, suggesting that they are important exploration values as potential targets in future studies.

### 4.5 Cross-ethnic comparative analysis of ARHL candidate genes

Among the 210 ARHL candidate genes integrated in this study, 56 genes were repeatedly reported across studies involving different geographic populations, forming a cross-ethnic network of gene associations. This finding suggests that despite population-specific differences in genetic background and environmental exposure, there exists a shared genetic foundation for ARHL across global populations. Overall, the data reveal both interregional gene overlap and clear geographical specificity. Notably, the highest number of reported candidate genes originates from studies in European populations, followed by North American and East Asian cohorts. This likely reflects not only regional disparities in research investment, but also differences in population genetic architecture that may influence gene detection rate and replicability across studies (Beaty et al., 2005). Moreover, the high degree of gene overlap between European and North American populations may in part reflect their shared ancestral heritage, resulted from the historical spread of European immigrants.

In this study, we noted a significant geographical and ethnic bias in the existing literature, with the majority of research samples concentrated on European populations, which limited the generalisability of the findings. However, several candidate genes have been repeatedly identified across independent studies in multiple ethnic groups, demonstrating strong cross-ethnic consistency. *CDH23*, *ILDR1*, and *SLC26A5* have emerged as high-confidence ARHL candidate genes in European, North American, and East Asian cohorts. These genes were derived from well-established auditory functional genes or monogenic deafness loci. Their consistent associations across different ethnic groups suggested their potential significance in the process of auditory aging. Therefore, despite the overall limited generalisability of evidence, some genes that were stably identified across populations may hold true global significance and may serve as essential components of future candidate gene panels.

*ILDR1*, initially identified as the causative gene for autosomal recessive non-syndromic hearing loss (DFNB42), has more recently been identified in ARHL. Hoffmann et al. (2016) first identified a common variant in *ILDR1* (rs2877561) significantly associated with ARHL and replicated the association in two large-scale cohorts: UK Biobank (European ancestry) and GERA (Non-Hispanic White). The variant showed consistent correlations with both speech reception thresholds (SRT) and speech discrimination scores (SDS). Subsequent linkage disequilibrium (LD) analysis of the *ILDR1* locus revealed that rs2332035 exhibited strong LD (*r*^2^ > 0.8) with ten surrounding SNPs, forming a stable haplotype block widely present across 26 populations, with particularly consistent patterns in five European and four Latin American groups. Furthermore, LD signals involving rs12638492 were also observed in East Asian and South Asian populations, providing additional support for *ILDR1*’s transethnic relevance (Flores et al., 2023). *CDH23*, originally implicated in ARHL through the discovery of the *ahl* allele in mouse models, has been extensively studied across ethnic groups. Its association with ARHL has been validated in European (Wells et al., 2019), African (Bouzid et al., 2018), Korean (Kim et al., 2016), and Japanese (Usami et al., 2022) cohorts. However, no significant association was detected in a Han Chinese cohort (Hwang et al., 2012), which may reflect to the fact that the detected sites were not key functional variants or insufficient sample size. Similarly, *SLC26A5* has been repeatedly reported across diverse ethnic groups in ARHL studies. Evidence from multi-ethnic genome-wide association analyses suggested that *SLC26A5* may lie near a genomic region linked to hearing decline with age (Lewis et al., 2022).

Beside cross-ethnic consistency, this study also highlighted ethnic-group-specific differences. Genetic variants that showed significant associations in one population may not replicate in others. Such specificity is not uncommon in the genetics of complex traits and reflects how genetic background modulates variant effect sizes across populations. A notable example is the *GRHL2* gene. It was initially identified in a multi-centre study across Europe, where multiple SNPs showed significant association with ARHL and demonstrated a consistent direction of effect across nine European cohorts (Van Laer et al., 2008). However, subsequent studies in East Asian populations failed to replicate these findings. Specifically, a study by Lin Y. H. et al. (2011) in an elderly Han Chinese cohort found no significant differences in the genotype distribution of *GRHL2* variants (e.g., rs10955255) between hearing-impaired and normal-hearing groups, offering no statistical support for an association between *GRHL2* and ARHL in this population. This result suggested the possibility that the same risk variant may exert differential effects across ethnic-groups, due to factors such as allele frequency differences, variations in linkage disequilibrium (LD) structure, or divergent gene-environment interactions. In addition to *GRHL2*, other risk loci have shown similar population-specific patterns. For instance, rs457717, a variant within the *IQGAP2* gene identified in European cohorts, was not significantly associated with ARHL in a Chinese male population (Luo et al., 2016), further supporting the presence of ethnicity-specific genetic effects in ARHL susceptibility.

In addition to somatic mitochondrial mutations, inherited mtDNA haplogroup differences may also influence individual susceptibility to ARHL. One such haplogroup, haplogroup U, was a common matrilineal lineage among Europeans and their descendants (e.g., North American Caucasians) (Mostafa et al., 2014). A study by Manwaring et al. (2007) found that individuals carrying haplogroup U had a significantly higher incidence of ARHL compared to other haplogroups. After adjusting for multiple covariates, haplogroup U remained independently associated with an increased risk of moderate-to-severe hearing loss, with a relative risk of approximately 1.63 (95% CI: 1.10–2.41). Subsequent research further indicated that individuals belonging to the haplogroup U might exhibit reduced mitochondrial oxidative phosphorylation function and elevated ROS levels, which could in turn diminish the antioxidant capacity of cochlear hair cells and accelerate the auditory aging process (Yasukawa et al., 2019). Although previous studies demonstrated that mtDNA haplogroups modulated the expression of nuclear genes such as SIRT3 (D’Aquila et al., 2012), and that haplogroup U was epidemiologically associated with an increased risk of ARHL, direct experimental evidence supporting synergistic interactions between haplogroup U and nuclear genes (e.g., *SIRT3* and *FOXO3*) in contributing to ARHL had not yet been sufficiently established.

In contrast, haplogroup A, a representative lineage prevalent in East Asians and their migratory descendants (such as Native American populations) (Alvarez-Iglesias et al., 2007), appeared to confer a different risk profile. A population-based study by Miura et al. in a Japanese cohort found that haplogroup A was significantly associated with increased risk of hearing loss in middle-aged men (aged 30–65), with an odds ratio of approximately 4.1 (*P* = 0.01). Meanwhile, another East Asian-predominant haplogroup, N9, was observed to exert a protective effect in women (Miura et al., 2022). This sex-specific difference suggested that the mtDNA-mediated energy metabolism pathway may have interacted with sex hormone levels to jointly influence the oxidative stress response of the auditory system (Huang et al., 2025).

### 4.6 High frequency candidate genes and demographic characteristics

#### 4.6.1 Relationship between genes and age

From congenital to 60 years old, patients with CDH23 gene mutations exhibited a wide range of onset ages for hearing loss. However, most cases showed congenital or early-onset hearing impairment, and shared a certain genotype–phenotype correlation (Miyagawa et al., 2012). In contrast, hearing loss caused by MYO6 gene mutations were generally progressive, with severity increasing with age. After the age of 40, the rate of hearing decline would accelerate significantly, averaging about 1.07 dB per year (Oka et al., 2020).

Mutations in the TECTA gene have a marked impact on the initial hearing threshold. Mutations in different structural domains (such as the ZP domain and the nidogen-like domain) corresponded to distinct audiogram configurations. However, unlike other deafness-related genes, the rate of hearing decline in individuals with TECTA mutations was roughly comparable to that of the normal population, suggesting that this gene primarily determines the starting point of hearing loss rather than affecting the age-related rate of auditory degeneration (Yasukawa et al., 2019).

Hearing loss associated with EYA4 gene mutations showed a broad age range of onset (5–61 years), though most patients develop symptoms after the age of 20. Longitudinal analyses indicated an average hearing decline rate of about 0.63 dB per year. Different mutation types produced distinct audiometric profiles: truncating mutations often lead to flat-type hearing loss, whereas non-truncating mutations tend to result in high-frequency loss (Shinagawa et al., 2020).

Additionally, specific single-nucleotide polymorphisms (SNPs) in the FOXO3 gene were closely associated with longevity. Individuals homozygous for the minor allele have a significantly lower mortality risk, suggesting that this gene may indirectly influence auditory aging by slowing age-related physiological degeneration (Ji et al., 2022).

#### 4.6.2 Relationship between genes and other demographic characteristics

In terms of population characteristics, research indicated that the SNP distribution of FOXO3 was not significantly associated with factors such as baseline age, sex, years of education, residence, marital status, exercise habits, smoking, drinking, or BMI (Ji et al., 2022). For other genes, existing studies primarily focused on genotype–phenotype correlations and the process of hearing change, with no sufficient evidence showing statistically significant associations between gene mutations and demographic variables such as sex, lifestyle, or socioeconomic factors.

## 5 Conclusion

Through bibliometric analysis and cross-ethnic comparison, this study highlighted both the rapid development and the structural limitations of research on the genetics of ARHL over the past three decades. Bibliometric results revealed an uneven global distribution of research efforts, with limited international collaboration, which constrained the integration and comparative analysis of multi-ethnic datasets. While the advent of GWAS and multi-omics approaches has led to the identification of numerous candidate genes, current research samples remained heavily skewed toward European and North American populations, with approximately 40% derived from European cohorts, and marked underrepresentation of East Asian, South Asian, African, and Latin American populations. Further analysis indicated that only 26% (56 out of 210) of the candidate genes have been replicated across two or more geographic populations, suggesting genetic heterogeneity and limitations of current findings in terms of mechanistic generalisability and clinical translation.

Overall, there is a pressing need to strengthen systematic genetic research in non-European populations, promote multi-ethnic joint analyses and data sharing, and establish a robust, cross-ethnically valid genetic risk framework. Such efforts will be essential for enabling individualised risk prediction and precision intervention strategies in ARHL.

## Data Availability

The original contributions presented in this study are included in this article/Supplementary material, further inquiries can be directed to the corresponding authors.
