# Supplementary material for: From genomic discovery to application in age-related hearing loss: a global bibliometric and cross-ethnic analysis
Source: Front Aging Neurosci. 2025 Nov 3;17:1678115. doi: 10.3389/fnagi.2025.1678115 (PMC12620367; doi:10.3389/fnagi.2025.1678115)
Supplement: Supplementary file 1 [file Table_1.docx]

*Supplementary Information*

**From Genomic Discovery to Application in Age-Related Hearing Loss：A Global Bibliometric and Cross-Ethnic Analysis**

Supplementary Table 1. Database Search Strategies......................................................................2

Supplementary Table 2. Inclusion & Exclusion Criteria..............................................................4

Supplementary Table 3. Core Journals Ranked by Number of Publications.......................4

Supplementary Table 4. Top 10 Key Genes in European Populations and Top 5 Functional Genes in Other Populations..........................................................................5

Supplementary Figure 1. Time-overlay visualisation of keyword co-occurrence in ARHL research (2010–2020).....................................................................................................................6

Supplementary Figure 2. Proportion of Study Population Distribution and Research Method Categories..........................................................................................................................................7

Supplementary Table 1. Database Search Strategies

| Databases | # | Concept | Search terms |
| --- | --- | --- | --- |
| **PubMed**  (Search conducted on: 2025.5.31) | 1 | Age-related hearing loss | presbycusis[MeSH Terms] OR ("hearing loss, sensorineural"[MeSH Terms] AND aged[MeSH Terms]) OR presbycusis[tiab] OR "age-related hearing loss"[tiab] OR ARHL[tiab] OR "age-induced hearing loss"[tiab] OR "elderly hearing loss"[tiab] OR (("hearing loss"[tiab] OR "hearing disorder*"[tiab] OR "hearing impairment"[tiab]) AND (aged*[tiab] OR aging*[tiab])) |
|  | 2 | Genetics and related terms | "Genes"[MeSH Terms] OR "Genetics"[MeSH Terms] OR "Genetic Techniques"[MeSH Terms] OR gene*[tiab] OR "candidate gene"[tiab] OR "functional gene"[tiab] OR "regulatory gene"[tiab] OR genetic*[tiab] OR DNA[tiab] OR cytogenetic*[tiab] OR genomic*[tiab] OR "medical genetic*"[tiab] OR "human genetic*"[tiab] OR heredit*[tiab] OR "Genome-Wide Association Stud*"[tiab] OR GWAS[tiab] OR genotype[tiab] OR epigenomics[tiab] OR SNP[tiab] OR "single nucleotide polymorphism"[tiab] OR "chromosome mapping"[tiab] |
|  | 3 | Final combined search | #1 AND #2 |
| **Web of Science Core Collection**  (Search conducted on: 2025.5.31) | 1 | Age-related hearing loss | TS=(presbycusis OR "age-related hearing loss" OR ARHL OR "age-induced hearing loss" OR "elderly hearing loss" OR (("hearing loss" OR "hearing disorder*" OR "hearing impairment") AND (aged* OR aging*))) |
|  | 2 | Genetics and related terms | TS=(gene* OR "candidate gene" OR "functional gene" OR "regulatory gene" OR genetic* OR DNA OR cytogenetic* OR genomic* OR "medical genetic*" OR "human genetic*" OR heredit* OR "Genome-Wide Association Stud*" OR GWAS OR genotype OR epigenomics OR SNP OR "single nucleotide polymorphism" OR "chromosome mapping") |
|  | 3 | Combined search | #1 AND #2 |
| **Embase**  (Search conducted on: 2025.5.31) | 1 | Age-related hearing loss | presbycusis/ OR (hearing loss, sensorineural/ AND aged/) OR presbycusis.ti,ab,kf. OR "age-related hearing loss".ti,ab,kf. OR ARHL.ti,ab,kf. OR "age-induced hearing loss".ti,ab,kf. OR "elderly hearing loss".ti,ab,kf. OR (("hearing loss" OR "hearing disorder*" OR "hearing impairment") AND (aged* OR aging*)).ti,ab,kf. |
|  | 2 | Genetics and related terms | genes/ OR genetics/ OR genetic techniques/ OR gene*.ti,ab,kf. OR "candidate gene".ti,ab,kf. OR "functional gene".ti,ab,kf. OR "regulatory gene".ti,ab,kf. OR genetic*.ti,ab,kf. OR DNA.ti,ab,kf. OR cytogenetic*.ti,ab,kf. OR genomic*.ti,ab,kf. OR "medical genetic*".ti,ab,kf. OR "human genetic*".ti,ab,kf. OR heredit*.ti,ab,kf. OR "genome-wide association stud*".ti,ab,kf. OR GWAS.ti,ab,kf. OR genotype.ti,ab,kf. OR epigenomics.ti,ab,kf. OR SNP.ti,ab,kf. OR "single nucleotide polymorphism".ti,ab,kf. OR "chromosome mapping".ti,ab,kf. |
|  | 3 | Combined search | #1 AND #2 |
| **Scopus**  (Search conducted on: 2025.5.31) | 1 | Age-related hearing loss | TITLE-ABS (presbycusis OR "age-related hearing loss" OR ARHL OR "age-induced hearing loss" OR "elderly hearing loss" OR (("hearing loss" OR "hearing disorder*" OR "hearing impairment") AND (aged* OR aging*))) |
|  | 2 | Genetics and related terms | TITLE-ABS (gene* OR "candidate gene" OR "functional gene" OR "regulatory gene" OR genetic* OR DNA OR cytogenetic* OR genomic* OR "medical genetic*" OR "human genetic*" OR heredit* OR "genome-wide association stud*" OR GWAS OR genotype OR epigenomics OR SNP OR "single nucleotide polymorphism" OR "chromosome mapping") |
|  | 3 | Combined search | #1 AND #2 |

Supplementary Table 2. Inclusion & Exclusion Criteria.

| **Inclusion Criteria** |
| --- |
| 1. The study focuses on age-related hearing loss (presbycusis). 2. The study reports results related to genetic factors (e.g., genes, polymorphisms, GWAS, etc.). 3. The study is designed as either original research or a review article. 4. The article is peer-reviewed and formally published. |
| **Exclusion Criteria** |
| 1. The article is a letter, erratum, editorial, or commentary. 2. The publication language is not in English. 3. The study has missing key information, full text is inaccessible, 4. The study is a duplicate publication. 5. The study population has hearing loss of similar nature caused by non-genetic factors. 6. The primary focus of the study is not the genetic basis of ARHL, or genetic factors are mentioned only as secondary or descriptive content. 7. The study focuses on syndromic hearing loss, where hearing loss is manifested in older age primarily driven by the syndrome. |

Supplementary Table 3. Core Journals Ranked by Number of Publications.

| Journals | Frequencies |
| --- | --- |
| HEARING RESEARCH | 40 |
| NEUROBIOLOGY OF AGING | 17 |
| ACTA OTO-LARYNGOLOGICA | 13 |
| PLOS ONE | 13 |
| FRONTIERS IN AGING NEUROSCIENCE | 10 |
| LARYNGOSCOPE | 10 |
| BRAIN RESEARCH | 9 |
| EUROPEAN JOURNAL OF HUMAN GENETICS | 9 |
| EXPERIMENTAL GERONTOLOGY | 9 |
| AUDIOLOGY AND NEURO-OTOLOGY | 8 |
| BIOCHEMICAL AND BIOPHYSICAL RESEARCH COMMUNICATIONS | 8 |
| PLOS GENETICS | 8 |
| SCIENTIFIC REPORTS | 8 |

Supplementary Table 4. Top 10 Key Genes in European Populations and Top 5 Functional Genes in Other Populations

| Ethnicity | Genes | Functions | Frequencies |
| --- | --- | --- | --- |
| Europe | EYA4 | Transcription Factors | 6 |
|  | MYO6 | Inner Ear Structure | 5 |
|  | NAT2 | Oxidative Stress Responses | 5 |
|  | GRM7 | Neurotransmitter Signaling | 5 |
|  | TRIOBP | Cytoplasmic Cytoskeleton | 4 |
|  | KCNQ4 | Ion Channels / Transporters | 4 |
|  | GRHL2 | Transcription Factors | 4 |
|  | COCH | Inner Ear Structure | 4 |
|  | SLC26A5 | Ion Channels / Transporters | 4 |
|  | GSTM1 | Oxidative Stress Responses | 4 |
| North America | GSTM1 | Oxidative Stress Responses | 3 |
|  | SLC26A5 | Ion Channels / Transporters | 2 |
|  | ISG20 | Inflammation & Immune Responses | 2 |
|  | TRIOBP | Cytoplasmic Cytoskeleton | 2 |
|  | ILDR1 | Inner Ear Structure | 2 |
| East Asia | CDH23 | Inner Ear Structure | 2 |
|  | GRM7 | Neurotransmitter Signaling | 2 |
|  | KCNQ4 | Ion Channels / Transporters | 2 |
|  | P2RX2 | Ion Channels / Transporters | 2 |
|  | ILDR1 | Inner Ear Structure | 2 |
| South America | ILDR1 | Inner Ear Structure | 2 |
|  | ISG20 | Inflammation & Immune Responses | 1 |
|  | TRIOBP | Cytoplasmic Cytoskeleton | 1 |
|  | SPTBN1 | Cytoplasmic Cytoskeleton | 1 |
|  | FXYD5 | Ion Channels / Transporters | 1 |
| South Asia | ILDR1 | Inner Ear Structure | 2 |
|  | GIPC3 | Inner Ear Structure | 1 |
|  | CLIC5 | Cytoplasmic Cytoskeleton | 1 |
|  | MYH14 | Cytoplasmic Cytoskeleton | 1 |
|  | COL9A3 | Cytoplasmic Cytoskeleton | 1 |
| Middle East | BAK1 | Mitochondrial Functions | 2 |
|  | BCL2 | Mitochondrial Functions | 2 |
|  | ESRRG | Transcription Factors | 1 |
|  | TJP2 | Inner Ear Structure | 1 |
|  | MT-RNR1 | Mitochondrial Functions | 1 |
| Africa | ILDR1 | Inner Ear Structure | 2 |
|  | TRIOBP | Cytoplasmic Cytoskeleton | 1 |
|  | COL5A1 | Cytoplasmic Cytoskeleton | 1 |
|  | ISG20 | Inflammation & Immune Responses | 1 |
|  | FXYD5 | Ion Channels / Transporters | 1 |


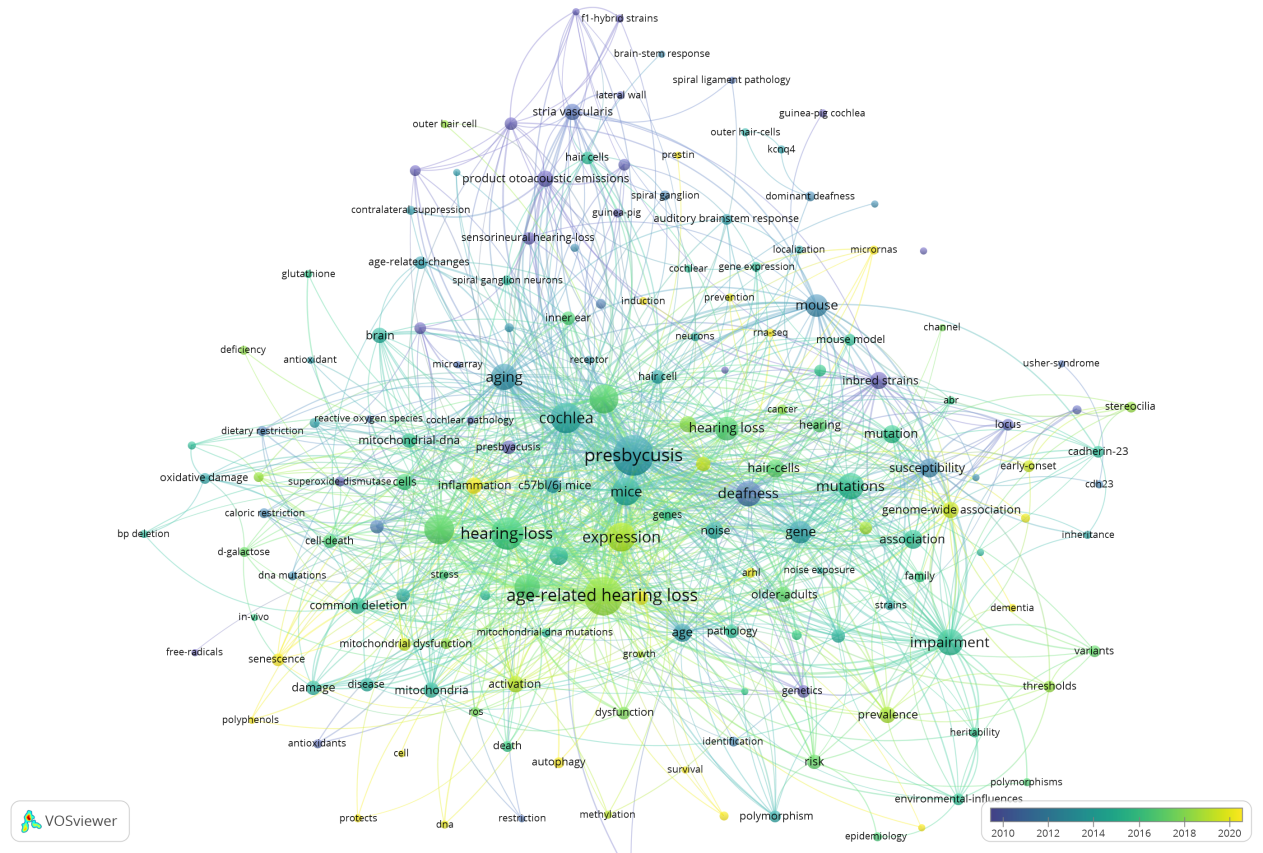


Supplementary Figure 1. Time-overlay visualisation of keyword co-occurrence in ARHL research (2010–2020)


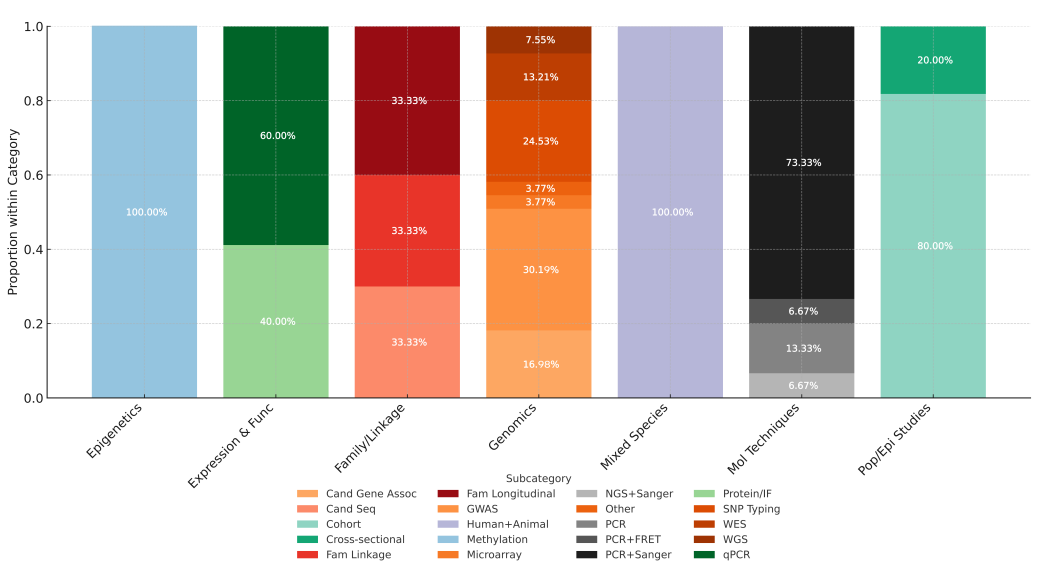

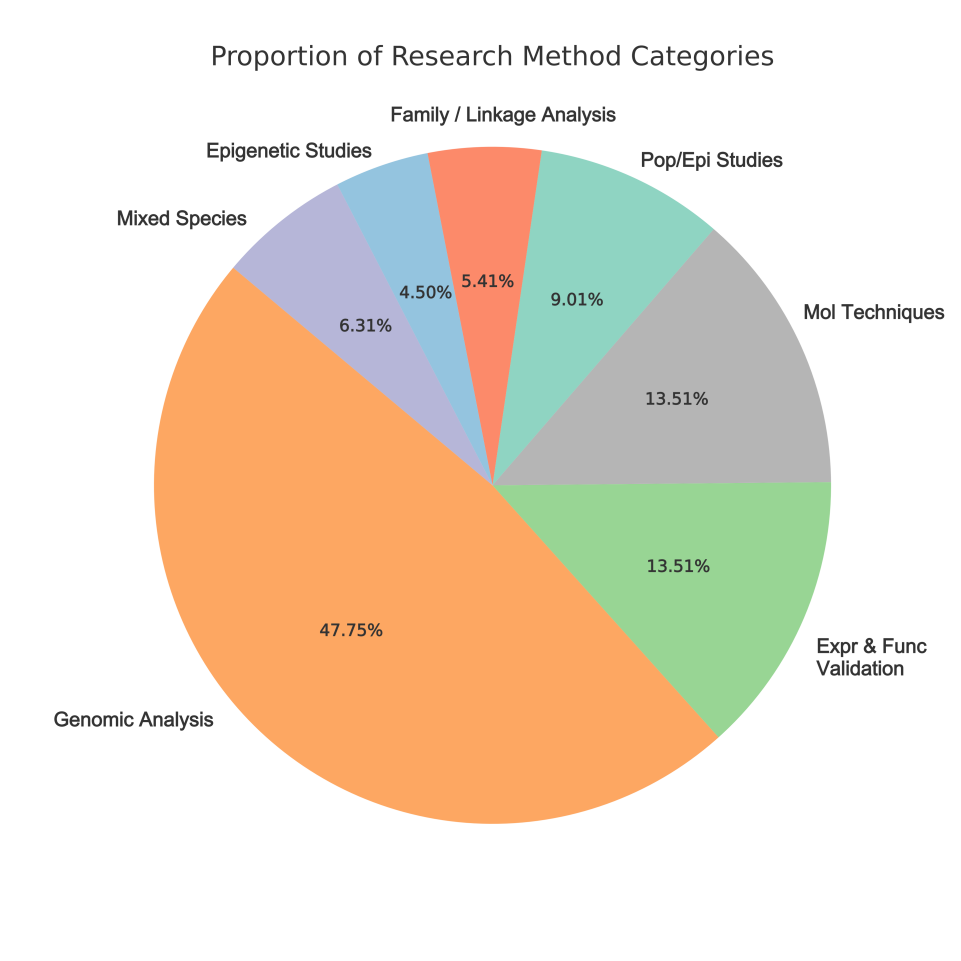

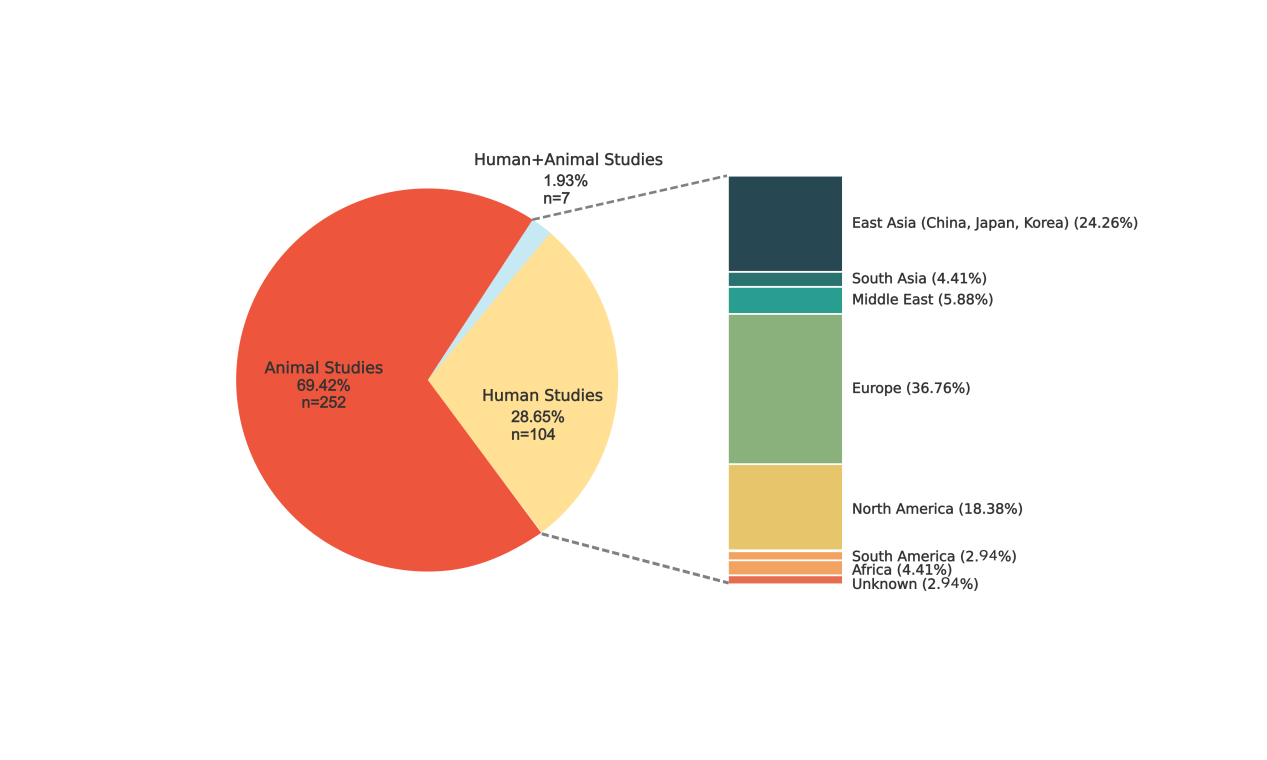


Supplementary Figure 2. Proportion of Study Population Distribution and Research Method Categories
